# Histologic, metabolomic, and transcriptomic differences in fir trees from a peri‐urban forest under chronic ozone exposure

**DOI:** 10.1002/ece3.11343

**Published:** 2024-05-13

**Authors:** Verónica Reyes‐Galindo, Juan P. Jaramillo‐Correa, Svetlana Shishkova, Estela Sandoval‐Zapotitla, César Mateo Flores‐Ortiz, Daniel Piñero, Lewis G. Spurgin, Claudia A. Martin, Ricardo Torres‐Jardón, Claudio Zamora‐Callejas, Alicia Mastretta‐Yanes

**Affiliations:** ^1^ Departamento de Ecología Evolutiva Instituto de Ecología, Universidad Nacional Autónoma de México Mexico City Mexico; ^2^ Programa de Maestría en Ciencias Biológicas Universidad Nacional Autónoma de México Mexico City Mexico; ^3^ Departamento de Biología Molecular de Plantas, Instituto de Biotecnología Universidad Nacional Autónoma de México Cuernavaca Morelos Mexico; ^4^ Jardín Botánico, Instituto de Biología Universidad Nacional Autónoma de México Mexico City Mexico; ^5^ Unidad de Biotecnología y Prototipos, Facultad de Estudios Superiores Iztacala Universidad Nacional Autónoma de México Tlalnepantla Estado de México Mexico; ^6^ School of Biological Sciences University of East Anglia Norwich Research Park Norfolk United Kingdom; ^7^ School of Biological Sciences The University of Edinburgh Edinburgh United Kingdom; ^8^ Departamento de Ciencias Ambientales Instituto de Ciencias de la Atmósfera y Cambio Climático, Universidad Nacional Autónoma de México Mexico City Mexico; ^9^ Bienes Comunales Santa Rosa Xochiac Mexico City Mexico; ^10^ Consejo Nacional de Humanidades, Ciencias y Tecnologías Mexico City Mexico; ^11^ Departamento de Ecología de la Biodiversidad Instituto de Ecología, Universidad Nacional Autónoma de México Mexico City Mexico

**Keywords:** *Abies religiosa*, natural settings, ozone pollution, terpenes, transcriptomics

## Abstract

Urbanization modifies ecosystem conditions and evolutionary processes. This includes air pollution, mostly as tropospheric ozone (O_3_), which contributes to the decline of urban and peri‐urban forests. A notable case are fir (*Abies religiosa*) forests in the peripheral mountains southwest of Mexico City, which have been severely affected by O_3_ pollution since the 1970s. Interestingly, some young individuals exhibiting minimal O_3_—related damage have been observed within a zone of significant O_3_ exposure. Using this setting as a natural experiment, we compared asymptomatic and symptomatic individuals of similar age (≤15 years old; *n* = 10) using histologic, metabolomic, and transcriptomic approaches. Plants were sampled during days of high (170 ppb) and moderate (87 ppb) O_3_ concentration. Given that there have been reforestation efforts in the region, with plants from different source populations, we first confirmed that all analyzed individuals clustered within the local genetic group when compared to a species‐wide panel (Admixture analysis with ~1.5K SNPs). We observed thicker epidermis and more collapsed cells in the palisade parenchyma of needles from symptomatic individuals than from their asymptomatic counterparts, with differences increasing with needle age. Furthermore, symptomatic individuals exhibited lower concentrations of various terpenes (ß‐pinene, ß‐caryophylene oxide, α‐caryophylene, and ß‐α‐cubebene) than asymptomatic trees, as evidenced through GC–MS. Finally, transcriptomic analyses revealed differential expression for 13 genes related to carbohydrate metabolism, plant defense, and gene regulation. Our results indicate a rapid and contrasting phenotypic response among trees, likely influenced by standing genetic variation and/or plastic mechanisms. They open the door to future evolutionary studies for understanding how O_3_ tolerance develops in urban environments, and how this knowledge could contribute to forest restoration.

## Graphical abstract

Urbanization alters ecosystems and evolutionary processes. Processes like air pollution by tropospheric ozone contribute to the decline of urban and peri‐urban ecosystems, such as *Abies religiosa* forests in the peripheral mountains southwest of Mexico City. Despite ozone exposure, some young individuals present minimal damage, suggesting contrasting phenotypic responses that may be influenced by genetic variations and/or plastic mechanisms, opening new perspectives for evolutionary studies on ozone tolerance and forest restoration.

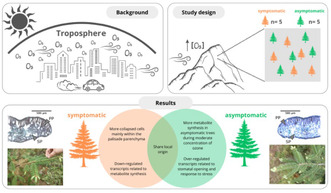

## RESUMEN

La urbanización altera tanto las condiciones del ecosistema como los procesos evolutivos, siendo la contaminación del aire, principalmente el ozono troposférico (O_3_), un factor que contribuye al declive de los bosques urbanos y periurbanos. Un ejemplo destacado son los bosques de oyamel (*Abies religiosa*) en las montañas periféricas al suroeste de la Ciudad de México, que han sufrido graves afectaciones por la contaminación de O_3_ desde la década de 1970. Resulta curioso observar que algunos individuos jóvenes presentan un daño mínimo relacionado con el O_3_ dentro de zonas con una exposición significativa a este contaminante. Aprovechando este entorno como un experimento natural, hemos comparado individuos asintomáticos y sintomáticos de edad similar (≤15 años; n = 10) mediante enfoques histológicos, metabolómicos y transcriptómicos. Las muestras de plantas se recolectaron durante días con concentraciones altas (170 ppb) y moderadas (87 ppb) de O_3_. Dado que se han llevado a cabo esfuerzos de reforestación en la región con plantas de diferentes poblaciones, primero confirmamos que todos los individuos analizados se organizaron dentro del grupo genético local en comparación con un amplio panel poblacional de esta misma especie (Análisis de Admixture con ~1.5 K SNPs). Observamos una epidermis más gruesa y más células colapsadas en el parénquima en empalizada de las agujas de los individuos sintomáticos que de sus contrapartes asintomáticas, y estas diferencias aumentaban con la edad de la aguja. Además, los individuos sintomáticos exhibieron concentraciones más bajas de varios terpenos (ß‐pineno, óxido de ß‐cariofileno, α‐cariofileno y ß‐α‐cubebeno) que los árboles asintomáticos, según se evidenció mediante GC–MS. Por último, los análisis transcriptómicos revelaron una expresión diferencial para trece genes relacionados con el metabolismo de carbohidratos, la defensa de plantas y la regulación génica. Nuestros resultados indican una respuesta fenotípica rápida y contrastante entre los árboles, probablemente influenciada por la variación genética presente y/o mecanismos plásticos. Estos hallazgos abren la puerta a futuros estudios evolutivos para comprender cómo se desarrolla la tolerancia al O_3_ en entornos urbanos y cómo este conocimiento podría contribuir a la restauración forestal.

## 1 INTRODUCTION

Rapid urbanization has severely disturbed entire ecosystems since the beginning of the industrial age (Bai et al., 2017), raising the important questions of how species cope with human‐transformed environments and which molecular, evolutionary, and ecological processes are involved (Rivkin et al., 2019). It is regularly thought that for species to persist in urban areas, they must adapt rapidly (Johnson & Munshi‐South, 2017). However, for adaptation to occur, selection needs to operate on heritable variation, which can determine whether a species persists or disappears from urban areas. Rapid adaptation seems particularly important for pollution tolerance, one of the strongest and most abrupt challenges that an urban species may face (Santangelo et al., 2018). This is especially challenging for long‐lived species, such as forest trees, implying that adaptation must occur within a few generations or be complemented by plastic responses (Müller‐Starck & Schubert, 2001). The genetic basis and plastic responses to pollution have been studied using a plethora of methods, from traditional provenance trials to genomic and transcriptomic analyses (Papadopulos et al., 2020; Whitehead et al., 2017). However, most research has been done under controlled conditions, meaning that studies in natural settings are needed for exploring the differential phenotypic responses in putatively tolerant versus sensitive individuals, and verifying if the same genes and pathways pinpointed in controlled studies can also be detected in the field.

One of the most common and harmful urban pollutants is tropospheric ozone (O_3_), which is generated by photochemical reactions that involve byproducts of fossil fuel burning (Churkina et al., 2017). Ozone is toxic to plants and has caused significant damage to forest ecosystems in and around heavily polluted cities (Ashmore, 2005; Cho et al., 2011). Given the key role that urban forests perform as providers of ecosystem services, understanding how O_3_ tolerance operates in trees is a pivotal step for informing conservation and reforestation programs of degraded (peri‐)urban forests. This requires field studies with an urban‐ecology perspective, aiming to understand how O_3_ tolerance develops and operates in natural settings, where tree responses to O_3_ are also expected to be more complex and entangled with other sources of stress (Nunn et al., 2006).

In plants, O_3_ damage, and the molecular mechanisms underlying the response to O_3_ exposure, has been studied for over 20 years, using both field and laboratory experiments with controlled conditions (Felzer et al., 2007; Hayes et al., 2020). O_3_ enters the plant through the stomata and triggers the formation of different reactive oxygen species (ROS), causing metabolic stress and resulting in cellular death, as ROS travel through the apoplast (Tausz et al., 2007). Several candidate genes have been postulated to cope with O_3_‐mediated metabolic stress (e.g., Hayes et al., 2020). However, strategies seem to differ between species and among populations within species (Baier et al., 2005; Hasan et al., 2021; Ludwików & Sadowski, 2008). For instance, differential sensitivity to ozone has been documented between poplars from more polluted and less polluted areas in the USA, according to both common garden and field experiments (Berrang et al., 1991). Furthermore, differential foliar damage (related to O_3_ exposure) has been observed among sacred fir (*Abies religiosa*) provenances in central Mexico (Hernández‐Tejeda & Benavides‐Meza, 2015).

**FIGURE 1:**
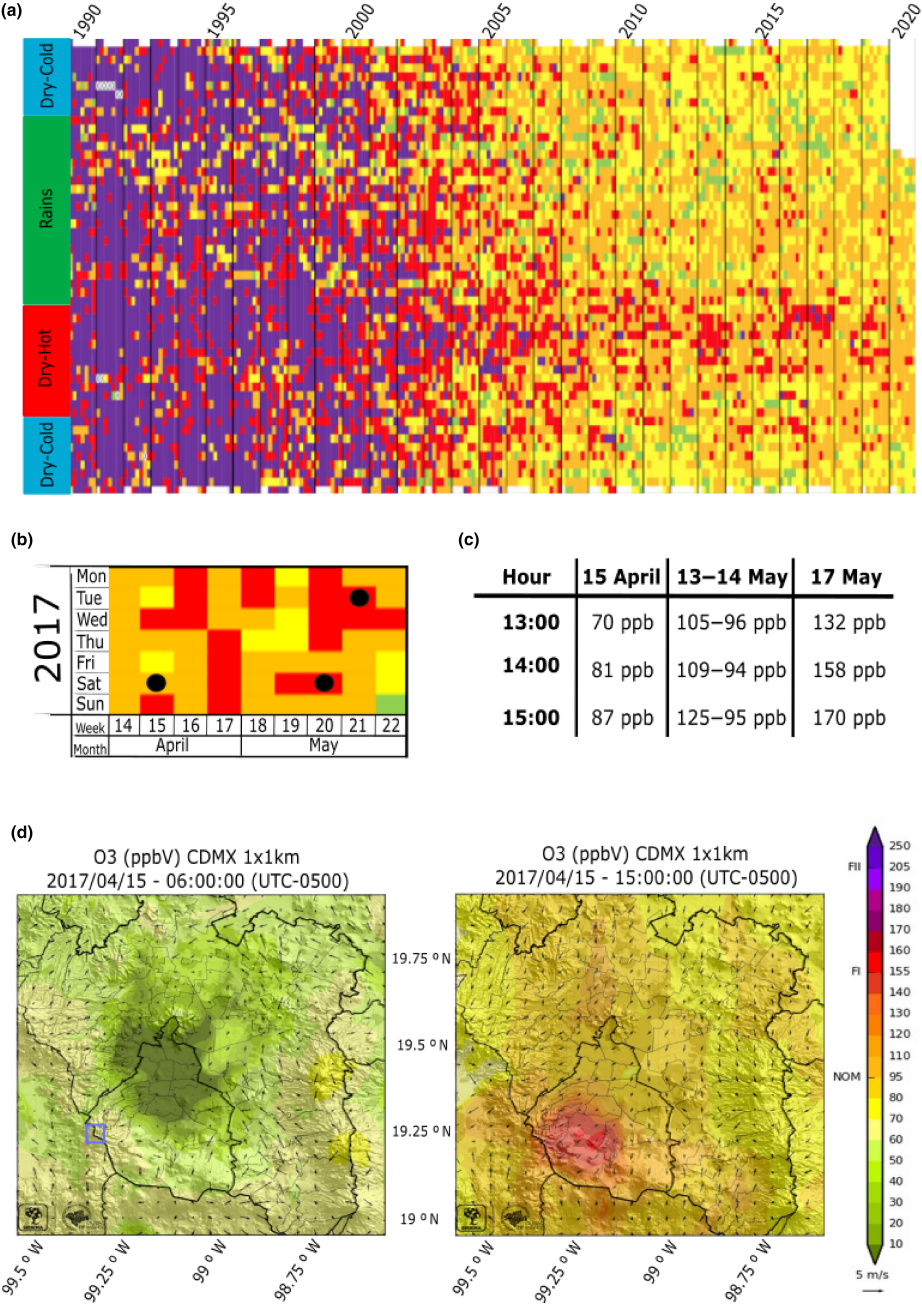
Change of O_3_ concentration in the Mexico City (CDMX) metropolitan area since 1990 (a) Air quality is represented by colors: green: good (0–70 ppb); yellow: regular (71–95 ppb); orange: bad (96–154 ppb); red: very bad (155–204 ppb), and purple: extremely bad (> = 205). Modified of Secretaría del Medio Ambiente (2020) (b) average O_3_ concentration during the study period (April and May, 2017). Black circles show collection days. (c) O_3_ concentration as measured at the nearby station to the sampling site (PEDREGAL) during the sampling period. Modified of SEDEMA (2018) (d) wind direction and O_3_ concentration in CDMX at 6:00 a.m. (~50 ppb; left) and at 3:00 pm (~130 ppb; middle; see colorimetric scale at right) on a regular day between April and May 2017. Blue boxes indicate the location of the study site. Arrow size indicates wind speed; vector at right (below colored bar) shows 5 m/s.

More than 5 million vehicles circulate daily in Mexico City (CDMX; INEGI, 2018), making it one of the most air‐polluted cities in the world (ONU, 2018). Its geographic location, mostly enclosed within a high‐elevation valley, and the high fossil fuel consumption generates perfect conditions for tropospheric O_3_ formation and accumulation (Bravo‐Alvarez & Torres‐Jardón, 2002; Molina et al., 2019). For instance, while O_3_ concentration in unpolluted air ranges between 20 and 50 ppb (Seinfeld, 1989), daily levels in CDMX continuously reached 200 ppb during the 1990s (Secretaría del Medio Ambiente, 2020; Figure 1a). Such elevated values still persist as isolated peaks (reaching up to 180 ppb by 2017; Secretaría del Medio Ambiente, 2020; Figure 1a), particularly between March and June, when temperatures in CDMX are the highest and precipitation the lowest (CONANP, 2006). Given that days with good air quality (i.e., <70 ppb) are still scarce (Figure 1a) and that O_3_ maxima are still well above the tolerable thresholds for human and ecosystem health (NOM‐020‐SSA1‐2104; Secretaría del Medio Ambiente, 2017), a constant selective force with strong episodic peaks, that coincide with the start of the growing season for most local plant species, is assumed to occur within the peri‐urban forests of CDMX.

Atmospheric drainage in CDMX mostly occurs between the southwestern mountains, which are dominated by sacred fir forests (Figure 1d; Alvarado‐Rosales et al., 2017). There is an ongoing decline of these forests, associated with the detrimental effects of O_3_ (de Bauer & Hernández‐Tejeda, 2007), inadequate management, excessive water extraction, and recurrent forest fires (Alvarado, 1989; Macías‐Sámano & Cibrían‐Tovar, 1989). Firs within these forests exhibit O_3_ damage in the form of reddish needles, which are rich in phenolic compounds and have degraded vacuoles and disintegrated spongy and palisade parenchyma (Alvarado‐Rosales & Hernández‐Tejeda, 2002; Alvarez et al., 1998). Damage becomes visible in 1‐year‐old needles, which die after the third year of exposure. When compared to unpolluted areas of the species' range, such damage often leads to decreased vigor and increased susceptibility to several pests (Alvarado‐Rosales & Hernández‐Tejeda, 2002; Hernández‐Tejeda & Benavides‐Meza, 2015).

Although previous studies have described O_3_ damage symptoms and pointed to this pollutant as the main cause for fir forest decline in CDMX (Alvarado, 1989; Alvarado‐Rosales & Hernández‐Tejeda, 2002; de Bauer & Hernández‐Tejeda, 2007), little attention has been paid to phenotypic differences for O_3_‐related symptoms until recently (Hernández‐Tejeda & Benavides‐Meza, 2015), when some apparently healthy young plants were observed within a heavily damaged stand. Complementing these observations in one of the most polluted cities of the world with methodological approaches to examine the effect of O_3_ on plants can improve our understanding of how O_3_ tolerance develops and operates in natural settings. For instance, at the histologic level, we could expect more cellular damage in symptomatic trees than in asymptomatic individuals. Similarly, a deficient regulatory response to the oxidative stress caused by O_3_ could be translated in the differential accumulation of certain metabolites, like some specific terpenes that have been observed in asymptomatic plants from various species after ozone exposure (Kopaczyk et al., 2020; Miyama et al., 2019). Lastly, transcriptomic analyses can help to narrow down the number of genes involved in the response to O_3_ exposure and to examine plastic responses in gene expression under varying levels of O_3_ (DeBiasse & Kelly, 2016).

Here, we explored the differential histologic, metabolomic (terpene), and transcriptomic responses to ozone pollution within a natural peri‐urban forest dominated by *A. religiosa*. Given that previous reforestation attempts have been carried out in this zone, we first determined the geographic origin of individuals and then looked for differentially expressed genes between asymptomatic and symptomatic trees during days of high and relatively low ozone concentrations. This study represents a first step to guide peri‐urban forest management from an eco‐evolutionary perspective.

## 2 MATERIALS AND METHODS

### 2.1 Study area and sampling

**FIGURE 2:**
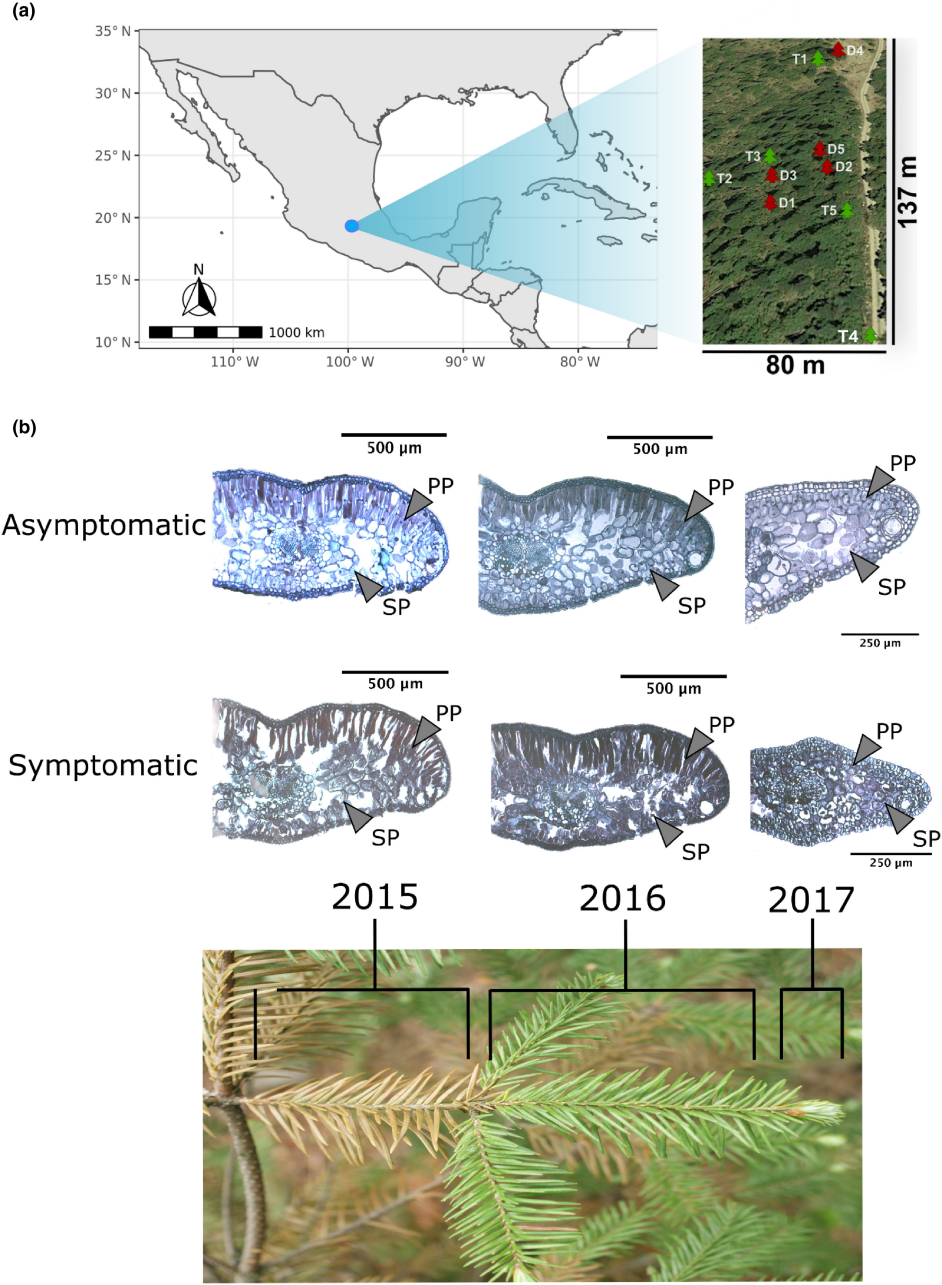
Distribution of focus trees (asymptomatic in green: T1–5; symptomatic in red: D1–5) within the study site, and location of the study site in Mexico (a) Transverse histological sections of needles from asymptomatic (top) and symptomatic (bottom) sacred fir individuals (*Abies religiosa*) for three growth periods (2015, 2016, and 2017) (b) Bars =250 and 50 μm. PP, palisade parenchyma; SP, spongy parenchyma.

The study site is located near CDMX, in one of the most exposed areas to tropospheric ozone, the “Cruz de Coloxtitla” ravine, in the village of Santa Rosa Xochiac, next to the “Desierto de los Leones” National Park (Alvarado‐Rosales et al., 2017; Figure 1d). We traced a quadrant of 80 × 137 m (19.285 N, −99.301 E; Figure 2a) within this zone and focused on young (10–15 years old) *Abies religiosa* ([Kunth] Schlechtendahl et Chamisso) trees. We chose five plants exhibiting large numbers of reddish needles, indicative of damage by O_3_ (Miller et al., 1994; hereafter referred to as “symptomatic” trees), as described elsewhere (Alvarado‐Rosales & Hernández‐Tejeda, 2002; Alvarez et al., 1998). Additionally, we selected five apparently healthy individuals, which had no visible damage in any branch (“asymptomatic” trees from hereon; Figure 2b, Appendix S1). Symptomatic and asymptomatic trees (*n* = 10) were distributed heterogeneously within the zone and were separated by at least 5 m from each other (Figure 2a). Needle samples were collected for each tree in three time points with contrasting O_3_ concentration: moderate (April 15, 2017; 87 ppb), intermediate (May 13–14, 2017, 120–94 ppb), and high (May 17, 2017; 170 ppb; Figure 1b,c), according to daily measurements from the nearest (PEDREGAL, PE) atmospheric station (available at http://www.aire.cdmx.gob.mx/default.php?opc=%27a8BhnmI=%27&opcion=Zg==). Needles were preserved in RNA Later and stored at −70°C until processing. The first sampling period roughly coincided with the start of the bud burst period for this population (personal observations). Sampling took place between 1:30 p.m. and 3:30 p.m. (Figure 1c); needles were selected from three sections of the same branch, in six branches per individual. Each branch section corresponded to a particular growth period (i.e., 2015, 2016, and 2017; Figure 2b). No symptomatic individual had leaves more than 3 years old.

### 2.2 Genotyping and geographic origin of tolerant trees

**FIGURE 3:**
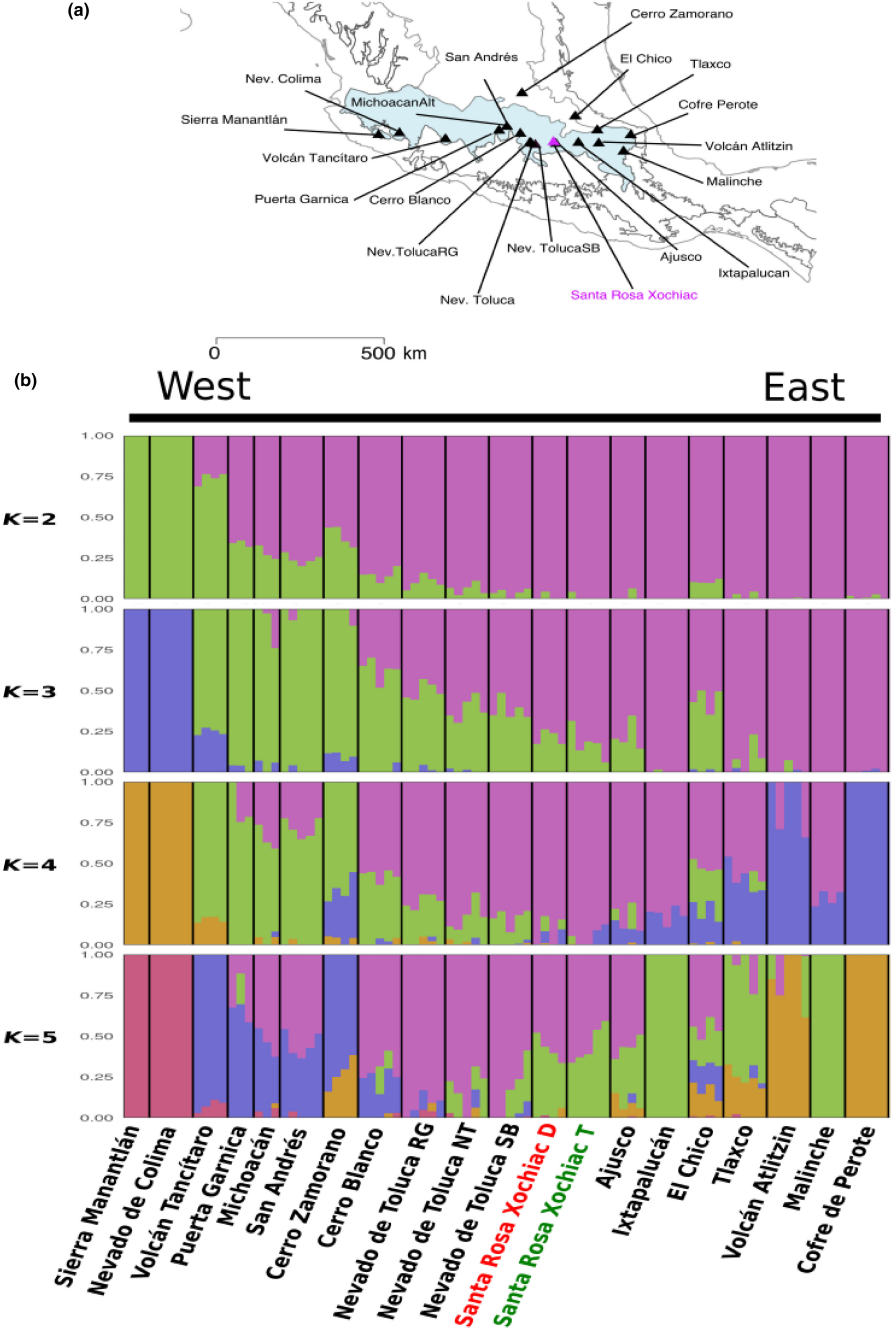
Assignment of studied individuals to the species genetic clusters based on admixture results (derived from 1550 SNPs). (a) Geoographic distribution of population samples included in the analyses. (b) Clustering of individuals as inferred with admixture when assuming between two and five genetic clusters (*k* = 2 to *k* = 5). Symptomatic trees indicated in red below figure and asymptomatic trees in green. Plots are shown for *k* = 2 to *k* = 5, all of which denote identical cluster assignments for both types of trees. Individuals (*n* = 88) are shown as vertical bars colored in proportion to their estimated ancestry for each cluster. Black lines separate populations listed from West to East along the species distribution (a).

Reforestation efforts in the study zone have involved germplasm from foreign provenances (Hernández‐Tejeda & Benavides‐Meza, 2015). To verify that sampled plants originated locally, from natural regeneration, we employed previously published SNP data for 318 individuals from 19 populations of *A. religiosa* distributed across its natural range (Giles‐Pérez et al., 2022). This data were used to assign the collected individuals to previously reported genetic clusters (Figure 3a). To do so, we used 80 mg of needle tissue for DNA extraction using liquid nitrogen and the QUIAGEN DNeasy® Plant Mini Kit (cat. No. 69104), following the manufacturer's protocol. DNA integrity was checked in 1% agarose gel, and its concentration quantified with a Qubit™ v 3.0. Libraries were prepared following the protocol from Poland and Rife (2012) after digestion with restriction enzymes *Msp*I (C | CGG) and *Pst*I (TGCA | G); a Pippin prep (SAGE sciences) was used to select the adequate fragment size before PCR amplification and sequencing. DNA sequencing was conducted in an Illumina‘s HiSeq2500 SE100 lane (100 bp) and in a Nextseq lane (100 bp) at the Institute of Integrative Biology and Systems at Université Laval, Canada (http://www.ibis.ulaval.ca/en/services‐2/genomic‐analysis‐platform/). Read quality was examined using FastQC (http://www.bioinformatics.braham.ac.uk/projects/fastqc/) before and after demultiplexing and quality filtering. Reads were assembled de novo, and ipyrad was used for SNP calling (Eaton, 2014). Parameters used were as follows: mindepth_statistical 8, mindepth_majrule 100,000, and clust_threshold 0.9. To optimize SNP calling, we followed the recommendations from Mastretta‐Yanes et al. (2015), modified for ipyrad. We aimed keeping SNPs genotyped in at least 90% of individuals and with minor allele frequencies (MAF) above 0.05. Individuals with more than 10% missing data were discarded with PLINK1.9 (Purcell et al., 2007), and additional random individuals were removed until retaining only 3–5 trees of each population, along with the 10 focus individuals of this study.

Pairwise relatedness between each pair of individuals within populations was calculated using PLINK 1.9 (Chang et al., 2015), as closely related individuals could bias further analyses, including population structure and assignment (Sethuraman, 2018). Only one of the focus (symptomatic) individuals was randomly discarded because of high relatedness (*r* > .25) with another symptomatic tree (Appendix S3). ADMIXTURE v 1.3.0 (Bhatta et al., 2019) was used to infer population structure by supposing between 1 and 5 genetic clusters (*K*); optimal *K* was assumed to be the one with smallest cross validation error (CV).

### 2.3 Anatomical analyses

Transverse histologic sections were prepared for five needles per branch from three branches of each tree, all sampled during the high O_3_ concentration periods. Following sampling, needles were embedded in distilled water according to Sandoval et al. (2005) and cut in 7–10 mm sections. Sections were immersed overnight in a fixative solution composed of 50% ethanol, 10% formaldehyde, 35% double distilled water, and 5% glacial acetic acid (FAA). After washing with distilled water and dehydration in a graded terbutylic alcohol series, the sections were embedded in Paraplast™, by adding 12–15 flakes every 30 min in an oven at 58°C, until doubling the alcohol volume. Sections were stored at 56°C for 3 weeks until forming solid blocks (inclusion cubes), which were further sectioned with a rotating microtome (American Optical 820; 12 μm). Ten to 15 transversal tissue sections were obtained per needle. The sections were first hydrated and dyed with safranin, then dehydrated within a graded ethanol series, and then stained with dye fast green (FCF), using a previously standardized method for sacred fir (Sandoval et al., 2005). Afterward, they were mounted on slides and dried for 15 days in an oven at 56°C. We looked for cell structures previously reported as symptoms of O_3_ damage (Appendix S2; Gimeno & Ibars, 2009). Samples were photographed in an Axioskope Car Zeiss photomicroscope for examining tissue‐level damage, compared to a reference description of *A. religiosa* (Alvarez et al., 1998).

### 2.4 Terpenes analysis

Two‐ and 3‐year‐old needles (corresponding to the growth years of 2015 and 2016) collected during moderate (87 ppb) and high (170 ppb) O_3_ concentration periods were used to quantify relative terpene abundances (Ibrahim et al., 2019). Approximately 80–95 mg (fresh weight) tissue preserved in liquid nitrogen was macerated with a mortar and pestle with 2 mL of dichloromethane, transferred to microtubes, and centrifuged (within tubes) for 1 min at 14,000 rpm. The supernatant was recovered and dried with compressed air, and the pellet was resuspended in 450 μL of dichloromethane and 50 μL of 1 mg/mL 1‐isopropylphenol (as internal standard). After homogenization, 2 μL were injected into a gas chromatograph with a Split/splitless injector (Agilent Technologies 6850 Network GC System) coupled to a mass spectrometer (5975C VL MSD with Triple‐Axis Detector) and a Xylan (Quadrex) 30 m × 0.25 mm × 0.25 μm capillary column. Analyses were performed at 230°C in the splitless mode (3 min). The initial temperature was set at 70°C for 2 min, then increased to 230°C at a rate of 20°C/min, and maintained for 5 min. Helium (i.e., carrier gas) was injected at a rate of 1 mL/min; the temperatures of the transfer line, ionization source, and quadrupole analyzer were 280, 230, and 150°C, respectively. Analyses were performed by electronic impact at 70 eV using the full spectrum scan mode (SCAN). For relative quantification, peak areas were integrated and normalized to the internal standard. Each peak (associated to a specific metabolite) was validated according to its retention time and mass spectrum based on the National Institute of Standards and Technology (NIST) library.

Only terpenes with similar fragmentation patterns or retention times (TR), observed in at least 60% of the samples and with at least 80% identification probability were retained. A matrix of relative abundance per 100 g of tissue was then generated for comparison between tree conditions (asymptomatic vs. symptomatic), periods (high and moderate O_3_), and needle age (2015, 2016; Figure 1) through a linear model using R (R Core Team, 2021), assuming a Gamma distribution. We compared the goodness of fit of the models with the Akaike's information criterion. The better model was Metabolites Concentration ∼ Condition * Period. We performed non‐paired comparisons, with Wilcoxon tests (Appendix S7), to explore variations in metabolite composition between asymptomatic and symptomatic groups, between periods (87 ppb vs. 170 ppb) and needle ages (1 year vs. 2 years). Analyses were performed in the stats package 4.1.2 (R Core Team, 2021) and results were visualized with ggplot2 3.3.5 (Wickham, 2016).

### 2.5 Differential expression analyses

One‐ and 2‐year‐old needles (2015 and 2016) sampled during the moderate (87 ppb) and high (170 ppb) O_3_ concentration periods were further analyzed for differential expression through RNA sequencing. Total RNA was isolated using a Spectrum RNA Plant™ kit (cat. No. STRN50, SIGMA) from 40 to 45 mg of tissue. RNA integrity was evaluated by 1% agarose gel electrophoresis, and its quality and purity were determined using NanoDrop (ultradifferential spectrophotometer) according to the 260/280 and 260/230 ratios. RNA concentration was quantified with a Qubit™ RNA IQ assay (Invitrogen). The 18 sequencing libraries from poly(A) + enriched RNA (Appendix S8) were prepared, and then sequenced in a Hi‐Seq 4000 in a 150PE sequencing lane at the University of Berkeley, USA (https://www.berkeley.edu/).

Demultiplexing was performed by the sequencing service. We performed quality checks with FastQC and removed adapters and low‐quality reads with Trimmomatic (Bolger et al., 2014) using the following parameters: ‐phred33, ILLUMINA CLIP: TruSeq3‐PE‐2.fa: 2: 30: 10, LEADING: 3, TRAILING: 3, SLIDING WINDOW: 10 MINLEN: 50. Reads were mapped to the *Abies balsamea* transcriptome (Van Ghelder et al., 2019; Bioproject PRJNA437248 in Genbank) with BWA‐MEM (Li & Durbin, 2009). Once the reads were mapped, we quantified the transcript abundance by counting the mapped reads per transcript for each sample (Appendix S9). Differential expression analyses were performed with DESeq2 (Love et al., 2014) and edgeR (Robinson et al., 2010) in R for the following comparisons: (1) symptomatic versus asymptomatic individuals during the high O_3_ concentration period (170 ppb); (2) asymptomatic trees during the moderate (87 ppb) versus high O_3_ concentration (170 ppb) periods; and (3) symptomatic individuals during the moderate (87 ppb) versus high O_3_ concentration (170 ppb) periods.

Transcripts with *p*‐values lower than .005, after fold change correction (Benjamini et al., 2001), were considered differentially expressed. Only those transcripts detected by both methods were retained and analyzed for identifying the most likely open reading frames. They were then annotated with TRAPID 2.0 (Van Bel et al., 2013) and BLASTx (https://blast.ncbi.nlm.nih.gov/Blast.cgi) using the nonredundant database (nr); we retained the first five hits for each transcript. For those transcripts that could not be annotated, we performed BLASTx searches against the Gymnosperm transcriptomes available at the Congenie database (congenie.org). Proteins of annotated transcripts were finally assigned to their respective metabolic pathways using KOALA (KEGG Orthology And Links Annotation) (Kanehisa et al., 2016).

## 3 RESULTS

### 3.1 Genotyping and geographic origin of trees

After de novo assembly and filtering, 1550 SNPs were genotyped for the 88 retained *A. religiosa* individuals distributed along most of its range (Giles‐Pérez et al., 2022), and for the 10 focus samples of this study. Although the optimal number of genetic clusters (*K*) for the Admixture analysis was 2, a higher value (*K* = 5) had a better resolution for differentiating groups in the eastern and western parts of the species distribution, allowing individual assignment. Both the symptomatic and asymptomatic trees of this study were assigned to the central‐Mexico cluster, to which trees from neighboring populations, such as Ajusco and Nevado de Toluca also belong (Figure 3). This result indicates that only local germplasm was included in our study.

### 3.2 Anatomical differentiation

Tissue differences were found between symptomatic and asymptomatic trees and among growth years (i.e., needles developed in 2015 and 2016 and sampled in 2017) within individuals (Figure 2b, Appendix S2). Needles of symptomatic trees exhibited a thicker epidermis and more collapsed cells than those of the asymptomatic ones, mainly within the palisade parenchyma (Figure 2b). In contrast, the spongy parenchyma, resin channels, and vascular tissues looked similar in the needles of symptomatic and asymptomatic individuals. Cell collapse became more evident with needle age in symptomatic trees (i.e., higher for 2015 than for 2016 needles), while asymptomatic individuals showed less cell collapse in the 2‐year‐old needles (2015) than in the 1‐year‐old needles (2016; Figure 2b).

### 3.3 Terpenes analysis

**FIGURE 4:**
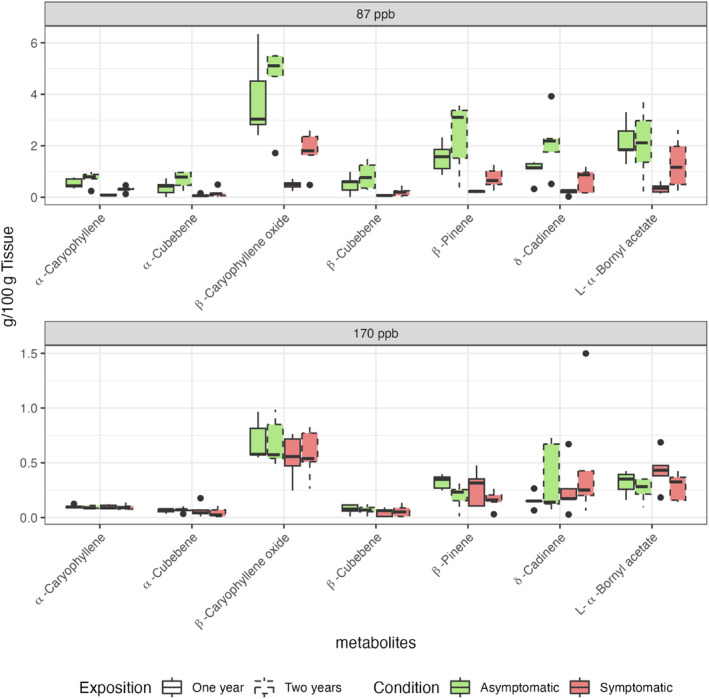
Relative sesquiterpene concentrations (mg/100 g dry weight) in needles from symptomatic (red) and asymptomatic (green) sacred fir (*Abies religiosa*) individuals during two periods with contrasting O_3_ concentration (87 ppb and 170 ppb). Measures taken from 1‐ (continuous line) and 2‐year old (dashed line) needles. Bars show variability in comparison to the IQR. See Table S4 to consult the statistical analyzes of interactions.

Compounds identified in all extracts included: ∂‐cadinene, α‐cubebene, ß‐cubebene, α‐caryophyllene, ß‐caryophyllene oxide, L‐α‐bornyl acetate, and ß‐pinene (Figure 4). The best model for explaining the differences in concentration of these shared terpenes (Nagelkerke's *R* ^2^ = .645), indicated an association with the tree's condition (symptomatic and asymptomatic) and the period of exposition (87 ppb vs. 170 ppb), with needle age being less relevant. Indeed, concentrations of all shared terpenes exhibited significant differences (*p* < .001, *p* < .01, or .05, Figure 4) between symptomatic and asymptomatic individuals during the period of moderate ozone concentration. In addition, there were statistical differences in the terpene concentrations of asymptomatic trees between periods (87 ppb vs. 170 ppb), but no differences were found between periods for the symptomatic trees or between needle ages (1 or 2 years).

### 3.4 Differential expression analyses (RNA‐seq)

**FIGURE 5:**
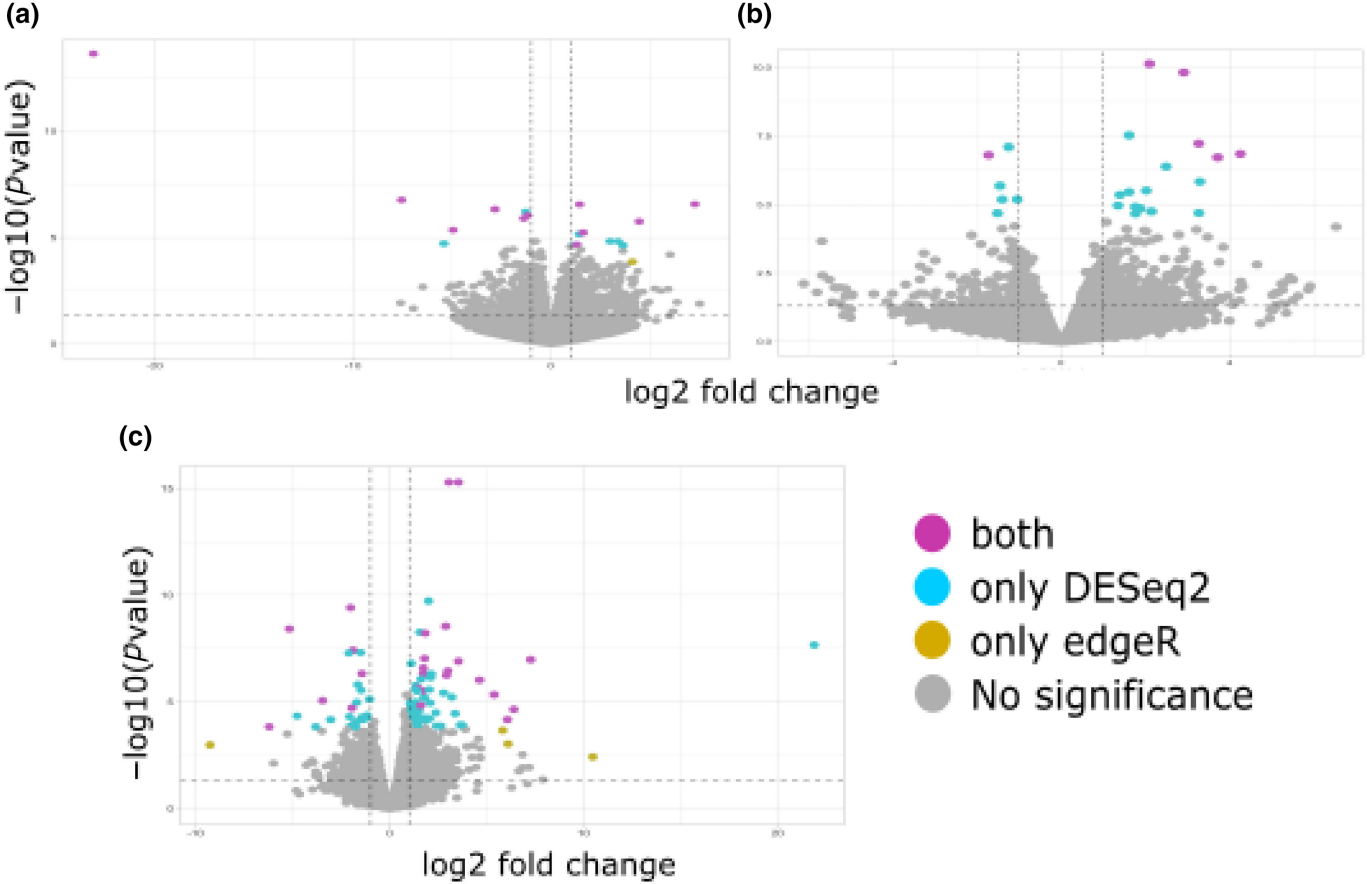
Differential expression analysis of RNA transcripts with two methods (DESeq2 in blue; edgeR in yellow; retained transcripts were those detected by both methods, in purple; *p* < .005). Volcano plots for asymptomatic versus symptomatic trees during the high O_3_ period (a); high versus moderate O_3_ concentration periods for symptomatic individuals (b); and high versus moderate O_3_ concentration periods for asymptomatic trees (c). Differentially expressed transcripts were selected with thresholds of fold change >2 (represented by two dotted black vertical lines) and *p* < .005 (represented by dotted black horizontal lines).

After quality filtering, 605,147,387 paired reads were retained for 18 samples, with an average of 33,619,299 reads per sample. The percent of reads mapped to the reference transcriptome (*A. balsamea*) ranged between 84.5% and 96.7% per sample (Appendix S9), indicating excellent transcript coverage. Eleven differentially expressed transcripts were identified in the needles of the symptomatic and asymptomatic trees (fold change) during the high O_3_ concentration period using both the DESeq2 and edgeR methods (Figure 5a). Five of them were upregulated and six were downregulated in asymptomatic individuals. Six of these transcripts could be annotated (Appendix S4) and were involved in carbohydrate metabolism, gene regulation, and defense, according to KOALA. All these transcripts belong to gene families whose members are involved in different aspects of abiotic and biotic stress response (see Appendix S4 for details), four of which have been previously associated with O_3_ response in controlled experiments with plants: *LRR receptor‐like protein kinases* (two annotated transcripts), an *L‐type lectin‐domain containing receptor kinase*, and a *chitinase* (Appendix S4).

When comparing transcript expression between trees with the same phenotype collected during low and high O_3_ concentration periods, we observed six and 22 differentially expressed transcripts for the symptomatic and asymptomatic individuals, respectively; 17 of which could be annotated (Figure 5b,c, Appendices S5 and S6). Remarkably, the number of differentially expressed transcripts in the asymptomatic plants was almost four times higher than that in symptomatic trees.

Among the five upregulated transcripts differentially expressed between periods in the symptomatic individuals, two transcripts were involved in the regulation of gene expression (encoding a *NAC* transcription factor and histone 1.3 variant) and one was involved in cell wall remodeling (encoding a *xyloglucan endotransglucosylase*). The only downregulated transcript for these symptomatic trees encoded an enzyme from the *UDP‐glucosyl transferase* family involved in various metabolic processes, including flavanol, tetrapyrrole, and terpene biosynthesis (Appendix S5). Homologs in other plant species for four of the upregulated transcripts have been previously associated with ozone response, including the abovementioned *NAC* transcription factor and UDP‐*glucosyl transferase* (Appendix S5).

For the asymptomatic trees, 16 of the 22 differentially expressed transcripts between periods could be annotated (Appendix S6). For two of them, no homologous amino acid sequences were found, but the results of BLASTn performed in the Congenie database suggest that these could, respectively, represent a conifer specific noncoding RNA, and a conifer‐specific peptide or protein. As for the annotated transcripts of these symptomatic individuals, they belong to gene families involved in response to abiotic and biotic stress, and the regulation of gene expression, four of these transcripts have been reported in controlled O_3_ experiments in plants (Appendix S6). Interestingly, these include the *linker histone H1*, which was also upregulated in the symptomatic trees during the high O_3_ concentration period.

## 4 DISCUSSION

In this study, we explored the histologic, metabolomic, and transcriptomic changes between symptomatic and asymptomatic fir trees within a natural population that has been heavily exposed to tropospheric O_3_ for over 40 years. According to our genetic ancestry analysis, all the studied individuals belong to the local gene pool, which suggests that the observed differences are the likely result of intrinsic evolutionary processes within this population. Such differences include histologic traits whose disparity increases with needle age, and contrasting terpene composition and gene expression. Our results illustrate how signals of O_3_ tolerance can arise in a natural population after a few decades of frequent exposure and shed light on the metabolic and gene regulation mechanisms involved in conifers.

### 4.1 Asymptomatic trees have a local genetic origin

Comparing the genetic ancestry of our focus trees with other populations allowed us to confidently assign them to the previously reported central‐Mexican genetic cluster (Giles‐Pérez et al., 2022; Figure 3b). This is important given that various reforestation efforts with foreign germplasm have been performed in the study zone and that some provenances have shown differential sensitivity to O_3_ (Hernández‐Tejeda & Benavides‐Meza, 2015). Given that reforested trees have still not reached reproductive maturity, O_3_ tolerance at the study site is the likely product of local processes, based on either plasticity or standing genetic variation (see below). Should genetic factors be involved, we hypothesize that only a relatively large effective population size could allow for the rapid evolutionary changes that are necessary to respond to such a strong environmental pressure in such a short term (1–2 generations if we consider a generation time of 25 years for sacred fir). Detailed quantitative and population genomics studies are thus necessary to evaluate tolerance heritability, estimate demographic parameters, and pinpoint the genomic bases of such putative adaptation.

### 4.2 Histologic O_3_ damage begins after only a few days of exposure

Overall, the symptoms observed herein were similar to those reported for other plants experimentally exposed to O_3_ under controlled conditions, at both the macroscopic and histologic levels (Chaudhary & Rathore, 2021; Moura et al., 2022). Such symptoms are different from those expected from other possible stresses, such as drought or disease, which produce yellowish needles and a more homogeneously affected foliage (including needle loss; Chastagner, 2001; Johnson et al., 2005). In contrast, in this study, the reddish needle symptoms indicative of O_3_ damage were first observed in 2‐year‐old needles, and foliage loss was limited to 3‐year‐old or older needles.

At the histologic level, the needles of all individuals bore signs of damage, albeit to a much lower degree for the asymptomatic trees than for the symptomatic individuals (Figure 2b, Appendix S2). This suggests a multivariate response to O_3_ exposure that results in a continuous rather than in a discrete phenotype, likely controlled by polygenic or epigenetic factors. Our data further shows that O_3_ damage begins at the tissue level during the first 30 days after bud burst (2017 buds; Figure 2b), even if symptoms are still not noticeable macroscopically. Such precocious signs have been described for other conifers, for which they could appear as early as the fifth day of exposure (Evans & Fitzgerald, 1993). Both the visible and histologic damages in firs aggravate with needle age (Figure 2), which indicates a cumulative and irreversible effect of O_3_ exposure (Schraudner et al., 1998), similar to that reported in controlled experiments in other plant species (Lee et al., 2020).

Cell collapse was particularly important within the palisade parenchyma (Figure 2b, Appendix S2; Alvarez et al., 1998; Evans & Fitzgerald, 1993; Terrazas & Bernal‐Salazar, 2002), which has been attributed to oxidizing agents that act on the middle lamella of the cell wall and promote its degradation (Gimeno & Ibars, 2009). Such degradation increases intercellular spaces and leads to cell death (Alvarez et al., 1998), and it is often accompanied by the accumulation of phenolic and tannin compounds that produce the characteristic reddish coloration of O_3_ damage (Figure 2b, Appendix S2; Gostin, 2010).

Symptomatic individuals had thicker epidermis than asymptomatic individuals (Ep; Figure 2b). Such thickening has already been associated with O_3_ response in conifers (Kivimäenpää et al., 2017) and might indicate increased synthesis of cell wall components under O_3_ stress (Sandermann et al., 1997). Interestingly, we did not find any differences in cuticle and resin duct structure between symptomatic and asymptomatic trees (Figure 2b, Appendix S2), which was reported as a recurrent sign of O_3_ damage in pines (Vollenweider et al., 2003). This suggests that either firs have a greater tolerance to O_3_ than pines or that such symptoms can only be observed when comparing individuals unexposed and exposed to O_3_ (which was impossible to settle in our study, because there are no zero‐exposure periods in the study site). However, our own casual field observations suggest that pines (i.e., *Pinus ayacahuite*, *P. hartwegii*, and *P. veitchii*) growing in the study site seem to be more affected than firs in terms of mortality, needle loss, and needle coloration.

### 4.3 Asymptomatic trees produce terpenes related to response to biotic and abiotic stress and recovery after stress

Changes in cell structure in ozone‐damaged plants may result from rampant oxidative stress (Baier et al., 2005; Iriti & Faoro, 2008). These may be produced by a deficient regulatory response, which results in the differential accumulation of certain metabolites, including terpenes (Kopaczyk et al., 2020; Miyama et al., 2019). Although we observed no clear anatomical differences in the resin ducts between symptomatic and asymptomatic trees, which could have indicated contrasting metabolite accumulation (Figure 4), there were significant differences in terpene composition, particularly sesquiterpenes, between asymptomatic and symptomatic phenotypes during the moderate O_3_ period. This is particularly compelling because sesquiterpenes, which were also found to increase their concentration in angiosperms when exposed to O_3_ (Kanagendran et al., 2018; Pellegrini et al., 2012), have been shown to degrade reactive oxygen species (ROS) and reduce cellular damage (Loreto & Fares, 2007; Vickers et al., 2009).

In our study, sesquiterpenes such as β‐pinene, δ‐cadinene, and β‐caryophyllene were observed at higher concentrations in the asymptomatic than the symptomatic trees prior to the high O_3_ concentration period (Figure 4a). Such compounds have been associated with antioxidant and larvicidal functions in several plant species, including pines (Govindarajan et al., 2016; Kanagendran et al., 2018; Loreto et al., 2004; Ortiz de Elguea‐Culebras et al., 2017). These terpenes could be allowing the asymptomatic trees to better cope with biotic and abiotic stresses once O_3_ exposure increases (Pellegrini et al., 2012). The whole biosynthetic pathway leading to these compounds should be of particular interest for future functional and evolutionary studies in firs and other plants. However, given that insects often attack already weakened trees (like those exposed to O_3_), such studies should also focus on disentangling the metabolic response to ozone exposure and insect defense.

Asymptomatic trees further produced a larger quantity of metabolites related to recovery after stress than symptomatic plants when we compared the metabolite composition between moderate and high O_3_ periods (Figure 4). Particularly β‐pinene, which has been previously related to the plant recovery after a high O_3_ exposure in *Nicotiana tabacum* (Kanagendran et al., 2018). This reinforces the idea that O_3_ exposure is the main cause of forest degradation at our study site.

The members of the family of *UDP‐glycosyltransferase* (UGT) enzymes participate in terpene biosynthesis (AB_008838_T.1; Appendix S5). The lower concentration of terpenes during the high O_3_ period (Figure 4) may be associated with the downregulation of these transcripts in symptomatic trees when comparing the low (87 ppb) and high (170 ppb) O_3_ concentration periods (Appendix S5). However, our study should be complemented by examining the concentration of other metabolites, like flavonoids or tannins, in the future. Indeed, our results indicated that the expression of transcripts involved in the flavonoid metabolic pathway could exhibit considerable differences compared with those found for terpene metabolism, as demonstrated by the transcriptomic data (AB_000811_T.1; Appendix S4). In any case, the metabolic signatures reported here could already be used to identify trees that are not adequately recovering after O_3_ exposure in affected forests.

### 4.4 Transcripts related to stomatal opening and response to stress are upregulated in asymptomatic trees

To further examine the molecular basis of O_3_ response, we performed a differential transcript expression analysis (DTE). We found differentially expressed transcripts when comparing asymptomatic and symptomatic trees during the high O_3_ concentration period (Appendix S4, Figure 5a) and when independently comparing concentration periods for individuals with the same phenotype (Appendices S5 and S6, Figure 5b,c). Homologs of several of these transcripts have been previously reported as differentially expressed in controlled O_3_ exposure experiments in angiosperms (Natali et al., 2018; Tammam et al., 2019; Waldeck et al., 2017), which suggests that the molecular mechanisms underlying response to O_3_ are conserved on a large evolutionary timescale.

The differentially expressed transcripts during high O_3_ concentration periods were associated with defense against pathogens and stomata opening, and included transcripts related to chitinases and LRR protein kinases. These proteins are known to play important roles in recognizing and responding to pathogens in plants (Vaghela et al., 2022; Wang et al., 2023), and their differential expression suggests either a response to an unaccounted pathogen attack (e.g., fungi) or that this signaling pathway is activated under both O_3_ exposure and other stressors. Again, this indicates the need for further studies to disentangling the response to O_3_ and biotic stress defense. Interestingly, some members of the LRR kinases gene family are also associated with the initial physiologic reaction of plants to O_3_ exposure, which involves stomatal closure (Hasan et al., 2021). Thus, studying stomata closure, and its underlying genes, should be a priority for future studies in natural plant populations affected by O_3_ pollution.

Comparing transcriptional profiles among trees with the same phenotype, asymptomatic or symptomatic, also showed differential responses to increased O_3_ concentration. In other words, the upregulated and downregulated transcripts belong to different GO categories. Among the upregulated transcripts in symptomatic individuals during the moderate O_3_ period (Figure 5b, Appendix S5), a homolog of the *xyloglucan endo‐*transglycosylase and a *non‐apical meristem* (NAM) transcription factor from the large NAC family stand out, as some of their homologs have been shown to play a key role in cell repair after O_3_ exposure (Zhang et al., 2017) and are activated by O_3_ during apoplastic ROS signaling (De Clercq et al., 2013). The activation of these pathways in symptomatic trees when O_3_ concentration is low, and might be indicative of decreased sensitivity to this pollutant when compared to the asymptomatic trees.

During the high O_3_ period, asymptomatic individuals upregulated some transcripts (Figure 5c, Appendix S6) related to plant resistance (NB‐ARC‐domain proteins), plant defense (peroxidases), and the flavonoid biosynthesis (chalcones) pathway (Dao et al., 2011; Krasensky et al., 2017). In other words, when O_3_ concentration increases, asymptomatic trees may be activating mechanisms related to stress response. Moreover, transcripts encoding for *UDP‐glycosyltransferase* (*UGT*) family members (Figure 5b, Appendix S5), which are essential components of the plant secondary metabolism pathway that helps detoxify harmful compounds (Pan et al., 2019), are downregulated in asymptomatic trees. *UGTs* are also essential for regulating various aspects of plant growth and development (Mateo‐Bonmatí et al., 2021).

All in all, the variety of pathways differentially activated between symptomatic and asymptomatic trees highlights the complexity of studying plant transcriptomic responses in natural conditions (Nunn et al., 2006). Indeed, several sources of stress are expected to act at the same time in degraded forests subjected to air pollution. To disentangle the various mechanisms involved, it is advisable to use controlled experiments, such as ozone top chambers (Abeyratne & Ileperuma, 2006; Palomäki et al., 1998), in combination with in situ studies in natural settings to understand how plants respond to stress under real‐life scenarios. However, although several sources of stress are at play in the peri‐urban forests of Mexico City, our histologic, metabolic, and transcriptomic analyses confirm that O_3_ pollution is an important stressor that triggers a rapid and differential phenotypic response in firs, likely modeled by standing genetic variation and/or plastic mechanisms. The evolutionary basis of such differences remains open to be explored. Since epigenetic variation is related to gene activity and expression (Richards et al., 2017; Srikant & Drost, 2021), and can accumulate faster than DNA mutations, their role in the phenotypic response to O_3_ pollution must be a priority for future studies.

## AUTHOR CONTRIBUTIONS

**Juan P. Jaramillo‐Correa:** Conceptualization (equal); funding acquisition (equal); methodology (equal); project administration (equal); resources (equal); supervision (equal); writing – original draft (equal). **Verónica Reyes‐Galindo:** Conceptualization (equal); data curation (lead); formal analysis (lead); methodology (equal); writing – original draft (equal). **Svetlana Shishkova:** Formal analysis (equal); methodology (equal); writing – review and editing (equal). **Estela Sandoval‐Zapotitla:** Methodology (equal); resources (equal); writing – review and editing (equal). **César Mateo Flores‐Ortiz:** Methodology (equal); resources (equal). **Daniel Piñero:** Resources (equal); writing – review and editing (equal). **Lewis G. Spurgin:** Methodology (equal); writing – review and editing (equal). **Claudia A. Martin:** Methodology (equal); writing – review and editing (equal). **Ricardo Torres‐Jardón:** Methodology (equal); writing – review and editing (equal). **Claudio Zamora‐Callejas:** Resources (equal). **Alicia Mastretta‐Yanes:** Conceptualization (equal); formal analysis (equal); funding acquisition (equal); methodology (equal); project administration (equal); resources (equal); software (equal); supervision (equal); writing – original draft (equal).

### OPEN RESEARCH BADGES

This article has earned an Open Data badge for making publicly available the digitally‐shareable data necessary to reproduce the reported results. The data is available at ASRA NCBI database (https://www.ncbi.nlm.nih.gov/sra/) deposited under the PRJNA1100728 Bioproject and the Dryad repository https://doi.org/10.5061/dryad.wpzgmsbw9.

## Supporting information


Appendices S1‐S9

## Data Availability

Histologic images and processed terpenes, genotype (vcf files) and transcriptomic (expression tables) data are available at the Dryad repository https://doi.org/10.5061/dryad.wpzgmsbw9. Pipelines and code for all analyses are available at the Github repository (https://github.com/VeroIarrachtai/Abiesreligiosa_vs_ozone). Transcriptome raw reads are available in the SRA NCBI database (https://www.ncbi.nlm.nih.gov/sra/) deposited under the PRJNA1100728 BioProject. Demultiplexed sequencing GBS data, including those samples previously analyzed in a phylogenetic survey (i.e., 80 samples, Giles‐Pérez et al., 2022), were deposited in NCBI with the Bioproject ID: PRJNA856692.
