# Supplementary material for: Histologic, metabolomic, and transcriptomic differences in fir trees from a peri‐urban forest under chronic ozone exposure
Source: Ecol Evol. 2024 May 13;14(5):e11343. doi: 10.1002/ece3.11343 (PMC11091488; doi:10.1002/ece3.11343)
Supplement: Supplementary file 1 — Appendices S1‐S9 [file ECE3-14-e11343-s001.zip › APPENDICES_EcologyEvolution_PEditor.docx]

**APPENDICES**


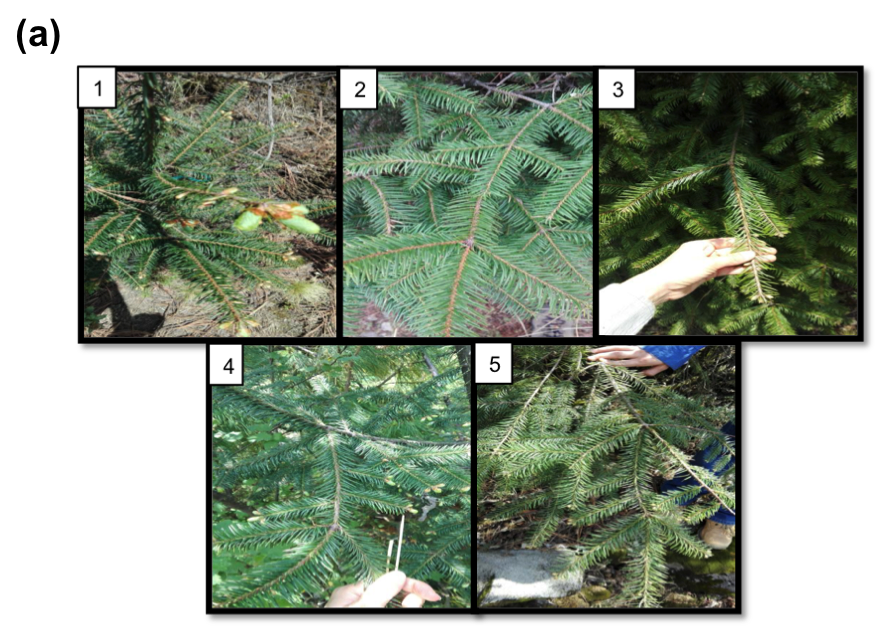


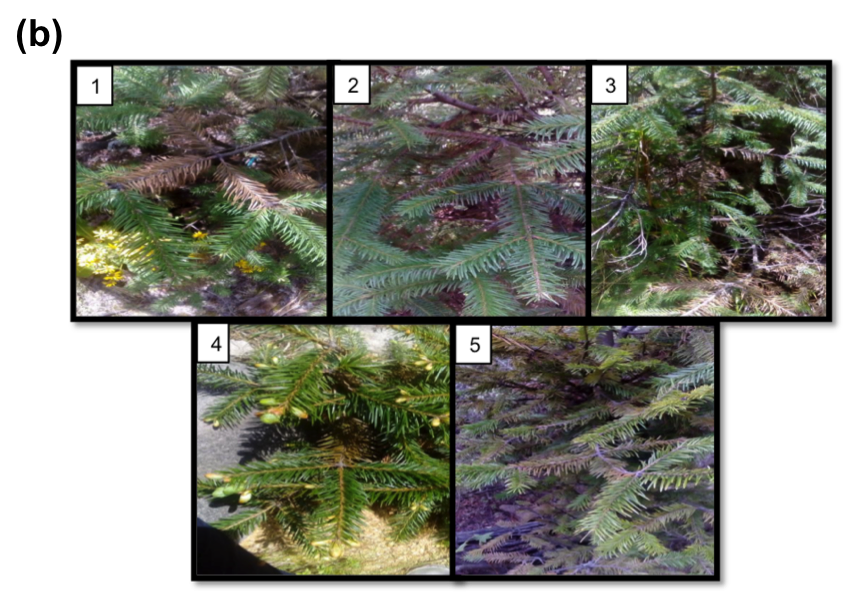


**Appendix S1** Photographs of the branches for each sampled sacred fir tree. **(a)** asymptomatic trees **(b)** symptomatic trees.


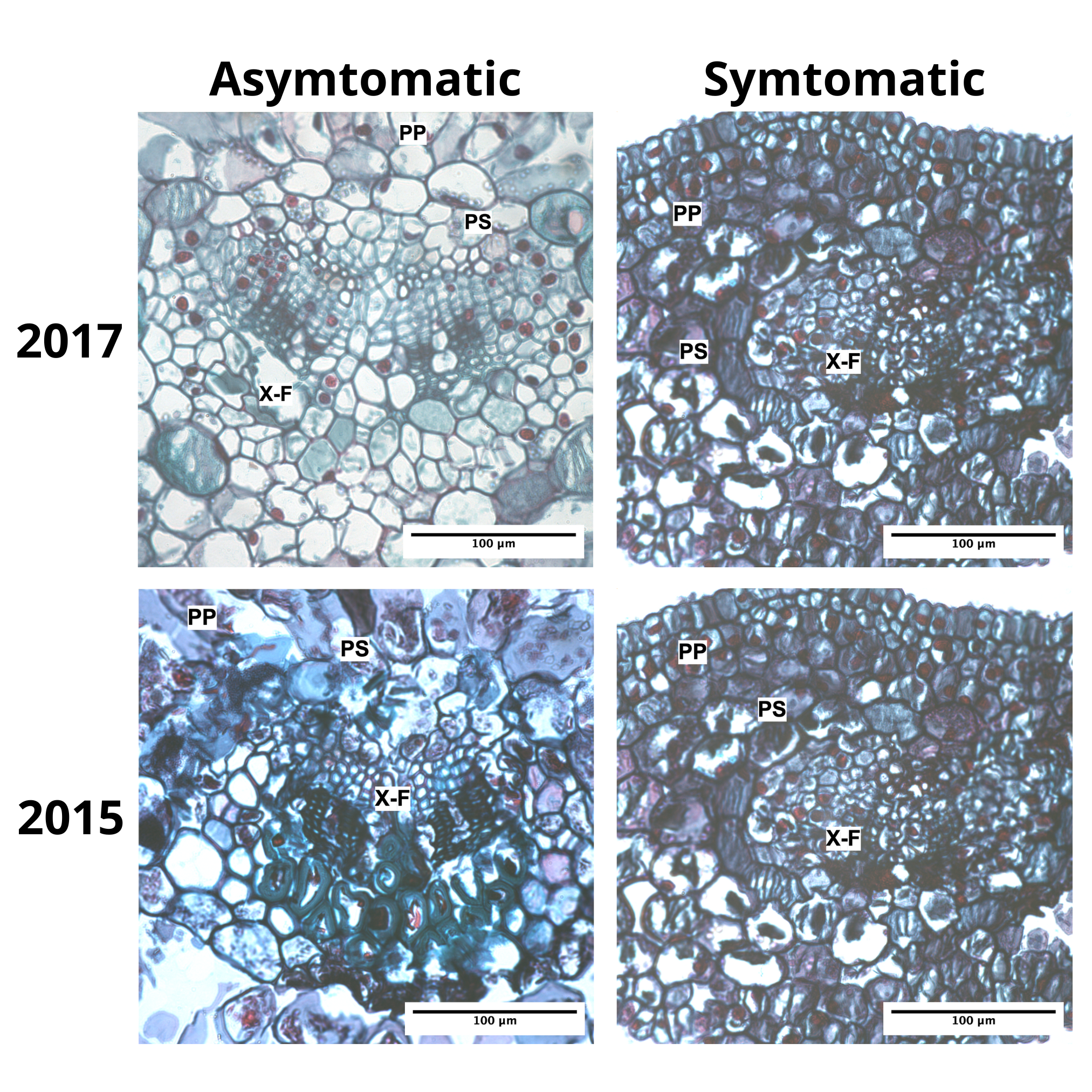


**Appendix S2** Histological sections of needles from asymptomatic (left) and symptomatic (right) sacred fir (*Abies religiosa*) individuals from two growing seasons (2017 top; 2015 bottom). All bars = 10µm. PP, palisade parenchyma; SP, spongy parenchyma; X-P, xylem and phloem.


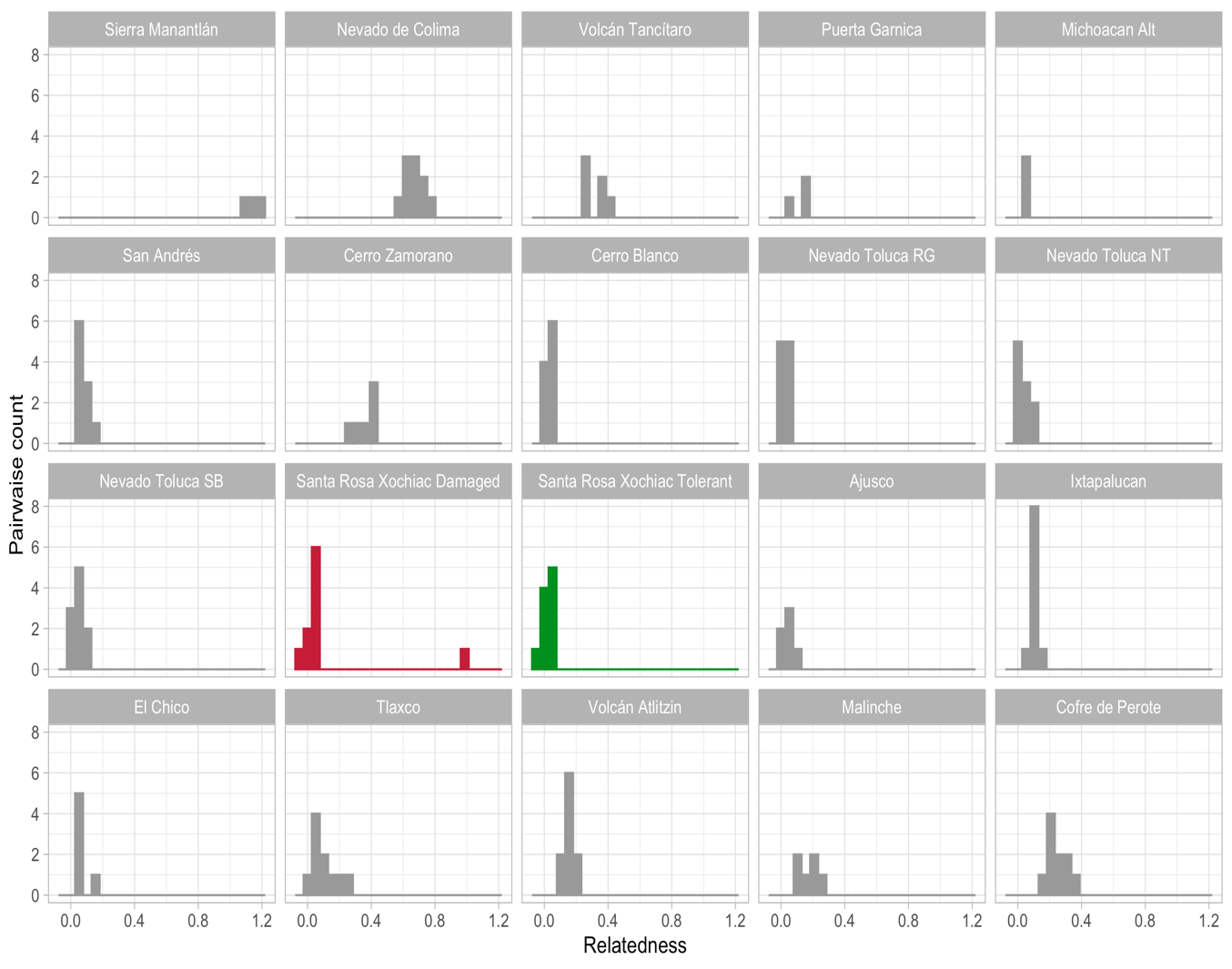


**Appendix S3** Relatedness between sacred fir (*Abies religiosa*) individuals used for genetic assignment analyses. Asymptomatic individuals from study sites in green, symptomatic trees in red.

**Appendix S4** Differentially expressed transcripts in symptomatic vs asymptomatic sacred fir (*Abies religiosa*)

| **Contig ID** | **Log_2_**  **fold change^a^** | **Query length, nts** | **Score^b^ / Max query cover in the 1st 5 hits, %** | **Annotation** | **Notes** |
| --- | --- | --- | --- | --- | --- |
| AB_000588_T.1 | 7.257 | 707 | L / 40 | Hypothetical protein KI387_017072, partial [*Taxus chinensis*] | The only hit returned by the ncbi Blastx |
| AB_045531_T.1 | 4.450 | 1192 | M / 68 | Hypothetical protein | Mostly bacterial hits |
| AB_015092_T.1 | 1.614 | 1944 | H / 89 | *Nuclear fusion defective 4-like, Nodulin-like* | *Nuclear fusion defective 4* in *A. thaliana* is involved in response to salt stress [(Sottosanto et al. 2007)](https://www.zotero.org/google-docs/?yzAC8o). |
| AB_036475_T.1 | 1.437 | 650 | H / 78 | *Chitinase* class VII / II / IV / or EP3-like / 4 / 5 | Chitinases are involved in responses to various abiotic and biotic stresses.  An acidic chitinase is **over-regulated after ozone exposure** in tobacco [(Ernst et al. 1992)](https://www.zotero.org/google-docs/?DGgOsC) |
| AB_018867_T.1 | 1.302 | 409 | L / 37 | Unknown [*Picea sitchensis*] | Four hits in 2 unknown proteins of *P. sitchensis* (Could be conifer-specific protein) |
| AB_029334_T.1 | -1.187 | 2594 | H / 72 | *Probable L-type lectin-domain containing receptor kinase S.5* | L-type lectin receptor kinases are involved in  defense response to bacteria and oomycetes [(Bouwmeester and Govers 2009)](https://www.zotero.org/google-docs/?pO82tp). |
| AB_029013_T.1 | -1.371 | 1214 | VL / 21 | Hypothetical protein | Three hits in two different OFRs |
| AB_035458_T.1  AB_038616_T.1 | -2.8306  - 4.951 | 928  752 | H / 99  H / 88 | *Leucine-rich repeat (LRR) receptor-like serine/threonine protein kinase* | A large family of LRR receptor-like kinases (RLK) participate in all aspects of plant development, in response to abiotic stresses, in defense processes and in plant-microbe interactions.  Loss of the LRR-RLK GHR1 **resulted in O3 sensitivity** in *A. thaliana*, likely mediated by the associated disruption of stomatal function [(Sierla et al. 2018)](https://www.zotero.org/google-docs/?VDGyNW). |
| AB_027319_T.1 | -7.549 | 895 | L / 39 | Tetratricopeptide repeat (TPR)-like / patatin-like phospholipase domain protein / oidium resistance required protein 1/ TOM1-like protein 2 | Members of TPR protein superfamily includes ones with potential to interact with Hsp90/Hsp70 as co-chaperones in nucleus and cytoplasm, thus participating in response to biotic stresses; RNA binding proteins involved in mRNA edition in plastid and mitochondria, are involved in plant development.  Patatin-like phospholipase domain proteins involved in plant development, synthesis of secondary metabolites, cell death, defense responses, response to abiotic stresses [(Lebeda et al. 2014)](https://www.zotero.org/google-docs/?fcwFuF). |
| AB_038562_T.1 | -23.104 | 951 | No hit | No hit | No significant similarity either in BLASTn search in NCBI nr database, neither in congenie. |
|  |  |  |  |  |  |

^a^ Positive value: up regulated in symptomatic trees; Negative value: down regulated in symptomatic trees;

^b^ H: high (>200); M: medium (80-200); L: low (50-80); VL: very low (40-50).

**Appendix S5** Differentially expressed transcripts in symptomatic sacred fir trees during high vs moderate O_3_ concentration periods.

| **ID Locus** | **Log_2_**  **fold change^a^** | **Query length, nts** | **Score^b^ / Max query cover in the 1st 5 hits, %** | **Annotation** | **Notes** |
| --- | --- | --- | --- | --- | --- |
| AB_002157_T.1 | 4.255 | 609 | VL / 30 | Hypothetical protein [*Acinetobacter baumannii*] | NCBI BLASTn returns five hits of mRNA sequences of *Picea glauca* with 81.49% to 87.93% identity |
| AB_028063_T.1 | 3.717 | 1034 | No hit | No hit |  |
| AB_029211_T.1 | 3.265 | 1193 | H / 44  (H / 76) | No Apical Meristem, (NAC) transcription factor  (Unannotated protein [*Picea sitchensis*]) | Members of the huge family of NAC transcription factors are involved in many aspects of plant development, defense response to bacteria and other organisms, response to water deprivation and to abscisic acid, secondary metabolic processes.  ANAC013, ANAC016, ANAC017, ANAC053 and ANAC078 regulate oxidative stress in *A. thaliana* [(De Clercq et al. 2013)](https://www.zotero.org/google-docs/?eeBfHV). |
| AB_023740_T.1 | 2.911 | 1320 | H / 62 | Xyloglucan endotrans  glucosylase (XET) /hydrolase;  Glycosyl hydrolase family 16 | XET enzymes participate in cell wall remodeling, thus modulating its expansion and strength.  The contig covers complete XET CDS.  Expression of XET coding gene XTR9 **increased in response to O_3_** [(Zhang et al. 2017)](https://www.zotero.org/google-docs/?HydwJb). |
| AB_015079_T.1 | 2.094 | 1291 | M / 24 | Linker histone H1 | Linker (H1) histones are the most variable histones; H1.3 variant of *A. thaliana* is involved in adaptive responses to abiotic stress [(Rutowicz et al. 2015)](https://www.zotero.org/google-docs/?3nXlOJ). |
| AB_008838_T.1 | -1.7 | 1312 | H / 89 | UDP-glucosyl transferase (UGT)  7-deoxyloganetin glucosyltransferase | The enzymes of the UGT family act on a variety of substrates and participates in many metabolic processes, including flavonol (e.g. UGT78D1/At1g30530), tetrapyrrole (e.g UGT85A1/AT1G22400) or terpenoid (e.g. UGT89B1/ AT1G73880) biosynthesis. Some UGTs involved in response to abiotic and biotic stresses [(Rehman et al. 2018)](https://www.zotero.org/google-docs/?sXKely).  Transcription of UGT78D2/At5g17050 gene was **decreased after O_3_ exposure** for 2 days [(Booker et al. 2012)](https://www.zotero.org/google-docs/?za1PyH). |

^a^ Positive value: up regulated during high O_3_ concentration periods; Negative value: down regulated during high O_3_ concentration periods;

^b^ H: high (>200); M: medium (80-200); L: low (50-80); VL: very low (40-50).

**Appendix S6** Differentially expressed transcripts in asymptomatic sacred fir trees during high vs. moderate O_3_ concentration periods**.**

| **ID Locus** | **Log_2_**  **fold change^a^** | **Query length, nts** | **Score^b^ / Max query cover in the 1st 5 hits, %** | **Annotation** | **Notes** |
| --- | --- | --- | --- | --- | --- |
| AB_010244_T.1 | 7.274 | 2007 | H / 59 | Metal tolerance protein (MTP) 5, 11  Cation diffusion facilitator (CDF) efflux family proteín | Plant MTPs from CDF family are involved in enhancing resistance to heavy metal tolerance |
| AB_022453_T.1 | 6.398 | 613 | M / 56 | Pathogenesis-related (PR) thaumatin family protein | PR thaumatin family proteins are involved in defense response, response to fungus, to osmotic stress, to water deprivation, to wounding, regulation of metabolism and plant development (e.g. AT4G36010 and AT1G20030 in *A. thaliana*). |
| AB_040533_T.1 | 6.07 | 561 | H / 90 | Disease resistance-responsive  dirigent-like protein | Many dirigent-like proteins are involved in defense response; some in response to wounding, cell wall biogenesis and metabolic processes. |
| AB_025629_T.1 | 5.388 | 1582 | H, M / 88 | LRR and NB-ARC domain disease resistance protein;  disease resistance protein RPP13, RPM1, RGA2, RGA4 | NB-ARC domain disease resistance (R) proteins in plants are involved in pathogen recognition and subsequent activation of innate immune responses. Besides, Glyma12g01420 was **upregulated in response to elevated ozone** in Glycine max [(Leisner et al. 2014)](https://www.zotero.org/google-docs/?psXoyd). |
| AB_022256_T.1 | 4.635 | 1436 | H / 82  -- | S-adenosyl methionine (SAM) synthase | Small family of plant S-adenosylmethionine synthases, or methionine  adenosyltransferase (MAT) produces SAM from methionine and ATP. Methyl group of SAM can be transferred to a variety of molecules that includes nucleic acids, proteins, lipids and secondary metabolites. Therefore, the methylation rates for a variety of substrates affects multiple aspects of plant fitness. Besides, in plants SAM is a precursor of ethylene and polyamines. Histone and DNA methylation is highly important for the regulation of gene expression [(Sekula et al. 2020)](https://www.zotero.org/google-docs/?RyGsRZ). |
| AB_013716_T.1 | 3.549 | 1989 | H / 74 | 3-ketoacyl (oxoacyl)-CoA synthase | Members of the 3-ketoacyl-CoA synthase family are involved in the biosynthesis of very long chain fatty acids (VLCFA), therefore, in cuticle development and wax and suberin synthesis. They also have an important role in response to cold, to light stimulus, to osmotic stress and to wounding |
| AB_043005_T.1 | 3.549 | 1193 | M / 63 | B-box-type Zinc finger and CCT domain protein CONSTANS-LIKE (COL) | COL transcription factors are involved in regulation of plant growth and development, control of flowering time and responses to stresses [(Khatun et al. 2021)](https://www.zotero.org/google-docs/?p6gd21). |
| AB_000610_T.1 | 3.054 | 1461 | H / 68 | beta-1,3-glucanase,  or glucan endo-1,3-beta-glucosidase | Beta-1,3-glucanases degrade plant callose and components of plant, fungi and bacteria cell walls, therefore, are involved in defense response. Some of them are also involved in response to cold, heat and wounding. |
| AB_021997_T.1 | 2.999 | 2144 | H / 81 | Isocitrate lyase/  Phosphoenolpyruvate phosphomutase | Isocitrate lyase is a glyoxylate cycle enzyme; it is involved in plant salt tolerance [(Yuenyong et al. 2019)](https://www.zotero.org/google-docs/?SzRuOR). |
| AB_002147_T.1 | 2.926 | 1211 | H / 82 | Peroxidase 72  class III peroxidase | *A. thaliana* Peroxidase 72 (AT5G66390) is involved in lignin biosynthesis and in response to oxidative stress; many class III peroxidases are located in cell wall and involved in cell wall modification; some may play a role in generating H_2_O_2_ during defense response.  **Near-ambient concentrations of ozone can induce ascorbate peroxidase** APX1 gene expression in *A. thaliana* and tobacco [(Kubo et al. 1995, Wang et al. 1999)](https://www.zotero.org/google-docs/?rkmD6k). At least part of the induction of heat shock proteins during light stress in Arabidopsis is mediated by H_2_O_2_ that is scavenged by APX1. |
| AB_000596_T.1 | 2.883 | 475 | No hit | No hit |  |
| AB_013152_T.1 | 1.832 | 1494 | H / 65 | Carboxylesterase 15;  alpha/beta hydrolase fold | Carboxylesterases hydrolyze esters of short-chain fatty acids and involved in metabolism of  jasmonic acid and salicylic acid and in systemic acquired resistance. They belong to the larger alpha/beta hydrolase fold superfamily of enzymes. |
| AB_028624_T.1 | 1.798 | 967 | H / 40 | Early nodulin-like (ENODL) with  cupredoxin/ plastocyanin domain | Cupredoxins contain type I copper centers and are involved in inter-molecular electron transfer reactions. ENODLs extracellular proteins are anchored in the plasma membrane. AtENODL1 (AT5G53870) transcript is up-regulated in leaves of *A. thaliana* subjected to a combination of drought and heat stress. AtENODL2 (AT4G27520) is involved on responses to water deprivation, abscisic acid, salt stress, light and temperature stimuli [(Rizhsky et al. 2004)](https://www.zotero.org/google-docs/?4jAbqZ). |
| AB_031334_T.1 | 1.736 | 752 | M / 40 | Zinc finger Ran-binding domain-containing protein 2; RNA-binding protein c17h9.04c; UPF0481 protein | Mammalian zinc finger Ran-binding domain-containing protein 2 is an RNA-binding protein involved in alternative splicing. |
| AB_015079_T.1 | 1.73 | 1291 | M / 24 | Histone H1 | Linker (H1) histones are the most variable histones; H1.3 variant of *A. thaliana* is involved in adaptive responses to abiotic stress [(Rutowicz et al. 2015)](https://www.zotero.org/google-docs/?SYeRyz). |
| AB_039330_T.1 | 1.601 | 974 | L (M) / 25 | Hypothetical protein (plants),  Set1 complex component ash2 | The Set1 complex specifically methylates Lys-4 of histone H3 (H3K4). H3K4me is an epigenetic modification involved in the **regulation (induction) of gene expression.** |
| AB_013119_T.1 | 1.429 | 465 | No hit |  | Two Picea NCBI BLASTn hits suggest that it could be conifer-specific polyA RNA. |
| AB_018867_T.1 | -1.431 | 409 | L / 37 | Unknown protein [*Picea sitchensis* only] | Could represent a conifer-specific protein |
| AB_000811_T.1 | -1.949 | 1592 | H / 61 | Flavonol synthase  2OG-Fe(II) oxygenase  GA2ox9, GA2ox10 | Some 2OG-Fe(II) oxygenases (as AT5G24530 in *A. thaliana*) participates in flavonoid biosynthesis; therefore, they may be involved in response to salicylic acid and defense response to bacteria, oomycetes and fungus.  A homology to GA2ox9 that contribute to cold  stress tolerance and involved in response to water deprivation and wounding [(Lange et al. 2020)](https://www.zotero.org/google-docs/?Bx4qbg), is also revealed. |
| AB_029470_T.1  AB_008960_T.1 | -3.459  -5.169 | 1182  1226 | H / 69  H / 80 | (Iso)eugenol synthase 1,  isoflavone reductase,  propenylphenol synthase 1  NmrA-like protein NAD(P)H-binding  NAD dependent epimerase/dehydratase family | The inferred proteins possess similarity to several classes of enzymes with Rossman fold. Among them are the isoflavone reductases involved in response to oxidative stress and to wounding, as well as the  propenylphenol synthases involved in synthesis of phenylpropanoid compounds, propenyl-phenols [(Wibe et al. 1997)](https://www.zotero.org/google-docs/?Sam86M), presumed to serve mainly in defense against herbivores and parasites. |
| AB_000071_T.1 | -6.206 | 1408 | H / 60 | Ferritin,  desiccation-related protein PCC13-62 | Arabidopsis ferritins are essential to protect cells against oxidative damage [(Ravet et al. 2009)](https://www.zotero.org/google-docs/?Pp7LdW). |

^a^ Positive value: up regulated during high O_3_ concentration periods; Negative value: down regulated during high O_3_ concentration periods;

^b^ H: high (>200); M: medium (80-200); L: low (50-80); VL: very low (40-50).

**Appendix S7** Wilcoxon Test. Interactions between Condition (asymptomatic or symptomatic), Needle age (2015 or 2016) and Period (high or moderate).

|  | **Period moderate** 87 ppb | | **Period high**170 ppb | |
| --- | --- | --- | --- | --- |
|  | **Metabolite** | **Sig.** | **Metabolite** | **Sig.** |
| **Condition**  Asymptomatic -  Symptomatic | α-caryophyllene | 0.0004871** | α-caryophyllene | N.S. |
|  | α-Cubebene | 0.007197* | α-Cubebene | N.S. |
|  | β-Caryophyllene | 0.0001299** | β-Caryophyllene | N.S. |
|  | β-Cubebene | 0.004525* | β-Cubebene | N.S. |
|  | β-Pinene | 0.0004871** | β-Pinene | N.S. |
|  | δ-Cadinene | 0.0007253** | δ-Cadinene | N.S. |
|  | Bornyl acetate | 0.0115* | Bornyl acetate | N.S. |
| **Needle age**  one-year and two-years exposition | α-caryophyllene | N.S. | α-caryophyllene | N.S. |
|  | α-Cubebene | N.S. | α-Cubebene | N.S. |
|  | β-Caryophyllene | N.S. | β-Caryophyllene | N.S. |
|  | β-Cubebene | N.S. | β-Cubebene | N.S. |
|  | β-Pinene | N.S. | β-Pinene | N.S. |
|  | δ-Cadinene | N.S. | δ-Cadinene | N.S. |
|  | Bornyl acetate | N.S. | Bornyl acetate | N.S. |

|  | **Metabolite** | **Sig.** |
| --- | --- | --- |
| **Period**  87ppb - 170 ppb | α-caryophyllene | 0.001953* |
|  | α-Cubebene | 0.003906* |
|  | β-Caryophyllene | 0.001953* |
|  | β-Cubebene | 0.003906* |
|  | β-Pinene | 0.001953* |
|  | δ-Cadinene | 0.005859* |
|  | Bornyl acetate | 0.001953* |

*(***) Significant at the 0.0001 probability level. (**) Significant at the 0.001 probability level. (*) Significant at the 0.05 probability level. (.) Significant at the 0.1 probability level. (ns) nonsignificant.*

**Appendix S8** Number of genes mapped for each sample.

| **Tree condition** | **O3 concentration period** | **ID sample** | **Number of genes identified as expressed**** | **Number of genes with no reads mapped*** |
| --- | --- | --- | --- | --- |
| Asymptomatic | high | Asymptomatic 1 | 37,601 | 0 |
|  |  | Asymptomatic 2 | 33,200 | 4,401 |
|  |  | Asymptomatic 3 | 34,182 | 3,419 |
|  |  | Asymptomatic 4 | 34,840 | 2,761 |
|  |  | Asymptomatic 5 | 33,366 | 4,235 |
|  | moderate | Asymptomatic 1 | 35,460 | 2,141 |
|  |  | Asymptomatic 2 | 34,256 | 3,345 |
|  |  | Asymptomatic 4 | 35,031 | 2,570 |
| symptomatic | high | Symptomatic 1 | 34,048 | 3,553 |
|  |  | Symptomatic 2 | 33,983 | 3,618 |
|  |  | Symptomatic 3 | 34,060 | 3,541 |
|  |  | Symptomatic 4 | 33,663 | 3,938 |
|  |  | Symptomatic 5 | 33,981 | 3,620 |
|  | moderate | Symptomatic 1 | 35,738 | 1,863 |
|  |  | Symptomatic 2 | 35,020 | 2,581 |
|  |  | Symptomatic 5 | 34,293 | 3,308 |

***Number of genes with no reads mapped**: refers to genes without any reads mapped to the reference transcriptome of *A. balsamea*, considering the total number of mapped genes.

** **Number of genes identified as expressed:** refers to genes with reads mapped to the reference transcriptome of *A. balsamea.*

**Appendix S9** RNA-seq data per sample.

| **Sample** | **Total**  **reads** | **Mapped** | **Mapped %** | **Properly paired** | **Properly paired %** | **Singletons** | **Singletons %** |
| --- | --- | --- | --- | --- | --- | --- | --- |
| Asymptomatic 1 | 26628465 | 25110645 | 94.30% | 23207744 | 87.79% | 190570 | 0.72% |
| Asymptomatic 2 | 29394389 | 27421473 | 93.29% | 25506062 | 87.47% | 216864 | 0.74% |
| Asymptomatic 3 | 28885822 | 26935913 | 93.25% | 25005412 | 87.24% | 206331 | 0.72% |
| Asymptomatic 4 | 27148620 | 24890979 | 91.68% | 23160294 | 85.90% | 190051 | 0.70% |
| Asymptomatic 5 | 25402180 | 22810050 | 89.80% | 21279266 | 84.36% | 153044 | 0.61% |
| Asymptomatic 1 | 86373044 | 80384008 | 93.07% | 74602376 | 87.09% | 601512 | 0.70% |
| Asymptomatic 2 | 39848295 | 36957834 | 92.75% | 34301814 | 86.78% | 271419 | 0.69% |
| Asymptomatic 4 | 30581813 | 28117524 | 91.94% | 26128276 | 86.06% | 188559 | 0.62% |
| Symptomatic 1 | 29917209 | 26626122 | 89% | 24575346 | 82.81% | 204199 | 0.69% |
| Symptomatic 2 | 20519755 | 19680381 | 95.91% | 18198494 | 89.39% | 124258 | 0.61% |
| Symptomatic 3 | 34920801 | 33514452 | 95.97% | 30677044 | 88.59% | 257139 | 0.74% |
| Symptomatic 4 | 33932229 | 30786857 | 90.76% | 28520838 | 84.73% | 245596 | 0.73% |
| Symptomatic 5 | 34662281 | 32472479 | 93.68% | 30328610 | 88.12% | 230530 | 0.67% |
| Symptomatic 1 | 29755812 | 25145836 | 84.51% | 23338336 | 79.07% | 219234 | 0.74% |
| Symptomatic 2 | 32034433 | 29891742 | 93.31% | 27696704 | 87.09% | 228013 | 0.72% |
| Symptomatic 5 | 39785361 | 35702980 | 89.74% | 32688214 | 82.84% | 330867 | 0.84% |

**Appendices references**

[Booker F, Burkey K, Morgan P, Fiscus E, Jones A (2012) Minimal influence of G-protein null mutations on ozone-induced changes in gene expression, foliar injury, gas exchange and peroxidase activity in Arabidopsis thaliana L.: Minimal influence of G-proteins on ozone responses. Plant Cell Environ 35:668–681.](https://www.zotero.org/google-docs/?EtVolC)

[Bouwmeester K, Govers F (2009) Arabidopsis L-type lectin receptor kinases: phylogeny, classification, and expression profiles. J Exp Bot 60:4383–4396.](https://www.zotero.org/google-docs/?EtVolC)

[De Clercq I, Vermeirssen V, Van Aken O, Vandepoele K, Murcha MW, Law SR, Inzé A, Ng S, Ivanova A, Rombaut D, van de Cotte B, Jaspers P, Van de Peer Y, Kangasjärvi J, Whelan J, Van Breusegem F (2013) The Membrane-Bound NAC Transcription Factor ANAC013 Functions in Mitochondrial Retrograde Regulation of the Oxidative Stress Response in Arabidopsis. Plant Cell 25:3472–3490.](https://www.zotero.org/google-docs/?EtVolC)

[Ernst D, Schraudner M, Langebartels C, Sandermann H (1992) Ozone-induced changes of mRNA levels of β-1,3-glucanase, chitinase and ‘pathogenesis-related’ protein 1b in tobacco plants. Plant Mol Biol 20:673–682.](https://www.zotero.org/google-docs/?EtVolC)

[Khatun K, Debnath S, Robin AHK, Wai AH, Nath UK, Lee D-J, Kim C-K, Chung M-Y (2021) Genome-wide identification, genomic organization, and expression profiling of the CONSTANS-like (COL) gene family in petunia under multiple stresses. BMC Genomics 22:727.](https://www.zotero.org/google-docs/?EtVolC)

[Kubo A, Saji H, Tanaka K, Kondo N (1995) Expression of arabidopsis cytosolic ascorbate peroxidase gene in response to ozone or sulfur dioxide. Plant Mol Biol 29:479–489.](https://www.zotero.org/google-docs/?EtVolC)

[Lange T, Krämer C, Pimenta Lange MJ (2020) The Class III Gibberellin 2-Oxidases AtGA2ox9 and AtGA2ox10 Contribute to Cold Stress Tolerance and Fertility. Plant Physiol 184:478–486.](https://www.zotero.org/google-docs/?EtVolC)

[Lebeda A, Mieslerová B, Petřivalský M, Luhová L, Špundová M, Sedlářová M, Nožková-Hlaváčková V, Pink DAC (2014) Resistance mechanisms of wild tomato germplasm to infection of Oidium neolycopersici. Eur J Plant Pathol 138:569–596.](https://www.zotero.org/google-docs/?EtVolC)

[Leisner CP, Ming R, Ainsworth EA (2014) Distinct transcriptional profiles of ozone stress in soybean (Glycine max) flowers and pods. :13.](https://www.zotero.org/google-docs/?EtVolC)

[Ravet K, Touraine B, Boucherez J, Briat J-F, Gaymard F, Cellier F (2009) Ferritins control interaction between iron homeostasis and oxidative stress in Arabidopsis. Plant J 57:400–412.](https://www.zotero.org/google-docs/?EtVolC)

[Rehman HM, Nawaz MA, Shah ZH, Ludwig-Müller J, Chung G, Ahmad MQ, Yang SH, Lee SI (2018) Comparative genomic and transcriptomic analyses of Family-1 UDP glycosyltransferase in three Brassica species and Arabidopsis indicates stress-responsive regulation. Sci Rep 8:1875.](https://www.zotero.org/google-docs/?EtVolC)

[Rizhsky L, Liang H, Shuman J, Shulaev V, Davletova S, Mittler R (2004) When defense pathways collide. The response of Arabidopsis to a combination of drought and heat stress. Plant Physiol 134:1683–1696.](https://www.zotero.org/google-docs/?EtVolC)

[Rutowicz K, Puzio M, Halibart-Puzio J, Lirski M, Kroteń MA, Kotliński M, Kniżewski Ł, Lange B, Muszewska A, Śniegowska-Świerk K, Kościelniak J, Iwanicka-Nowicka R, Żmuda K, Buza K, Janowiak F, Jõesaar I, Laskowska-Kaszub K, Fogtman A, Zielenkiewicz P, Tiuryn J, Kollist H, Siedlecki P, Ginalski K, Świeżewski S, Koblowska M, Archacki R, Wilczyński B, Rapacz M, Jerzmanowski A (2015) A specialized histone H1 variant is required for adaptive responses to complex abiotic stress and related DNA methylation in Arabidopsis. Plant Physiol:pp.00493.2015.](https://www.zotero.org/google-docs/?EtVolC)

[Sekula B, Ruszkowski M, Dauter Z (2020) S-adenosylmethionine synthases in plants: Structural characterization of type I and II isoenzymes from Arabidopsis thaliana and Medicago truncatula. Int J Biol Macromol 151:554–565.](https://www.zotero.org/google-docs/?EtVolC)

[Sierla M, Hõrak H, Overmyer K, Waszczak C, Yarmolinsky D, Maierhofer T, Vainonen JP, Salojärvi J, Denessiouk K, Laanemets K, Tõldsepp K, Vahisalu T, Gauthier A, Puukko T, Paulin L, Auvinen P, Geiger D, Hedrich R, Kollist H, Kangasjärvi J (2018) The Receptor-like Pseudokinase GHR1 Is Required for Stomatal Closure. Plant Cell 30:2813–2837.](https://www.zotero.org/google-docs/?EtVolC)

[Sottosanto JB, Saranga Y, Blumwald E (2007) Impact of AtNHX1, a vacuolar Na+/H+ antiporter, upon gene expression during short- and long-term salt stress in Arabidopsis thaliana. BMC Plant Biol 7:18.](https://www.zotero.org/google-docs/?EtVolC)

[Wang J, Zhang H, Allen RD (1999) Overexpression of an Arabidopsis Peroxisomal Ascorbate Peroxidase Gene in Tobacco Increases Protection Against Oxidative Stressl. :8.](https://www.zotero.org/google-docs/?EtVolC)

[Wibe A, Borg-Karlson A-K, Norin T, Mustaparta H (1997) Identification of plant volatiles activating single receptor neurons in the pine weevil (Hylobius abietis). J Comp Physiol A 180:585–595.](https://www.zotero.org/google-docs/?EtVolC)

[Yuenyong W, Sirikantaramas S, Qu L-J, Buaboocha T (2019) Isocitrate lyase plays important roles in plant salt tolerance. BMC Plant Biol 19:472.](https://www.zotero.org/google-docs/?EtVolC)

[Zhang L, Xu B, Wu T, Wen M, Fan L, Feng Z, Paoletti E (2017) Transcriptomic analysis of Pak Choi under acute ozone exposure revealed regulatory mechanism against ozone stress. BMC Plant Biol 17:236.](https://www.zotero.org/google-docs/?EtVolC)
