# Supplementary material for: Histologic, metabolomic, and transcriptomic differences in fir trees from a peri‐urban forest under chronic ozone exposure
Source: Ecol Evol. 2024 May 13;14(5):e11343. doi: 10.1002/ece3.11343 (PMC11091488; doi:10.1002/ece3.11343)
Supplement: Supplementary file 1 — Appendices S1‐S9 [file ECE3-14-e11343-s001.zip › Figures_Abies_EcologyEvolution-2.docx.pdf]

Supplementary Images

(a)

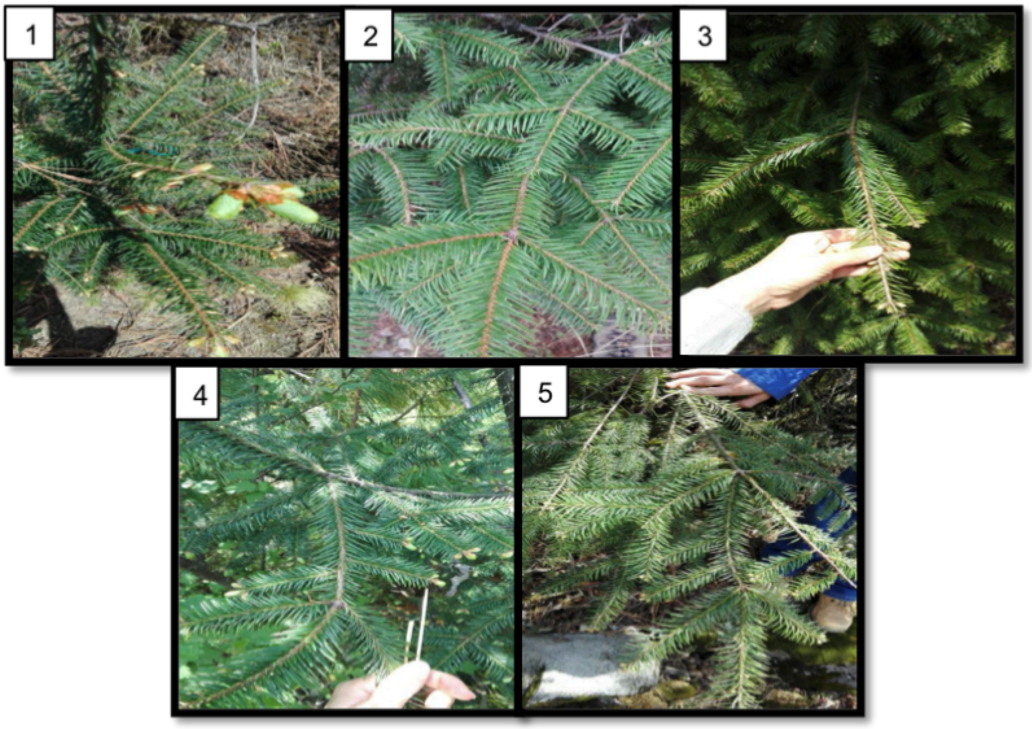

(b)

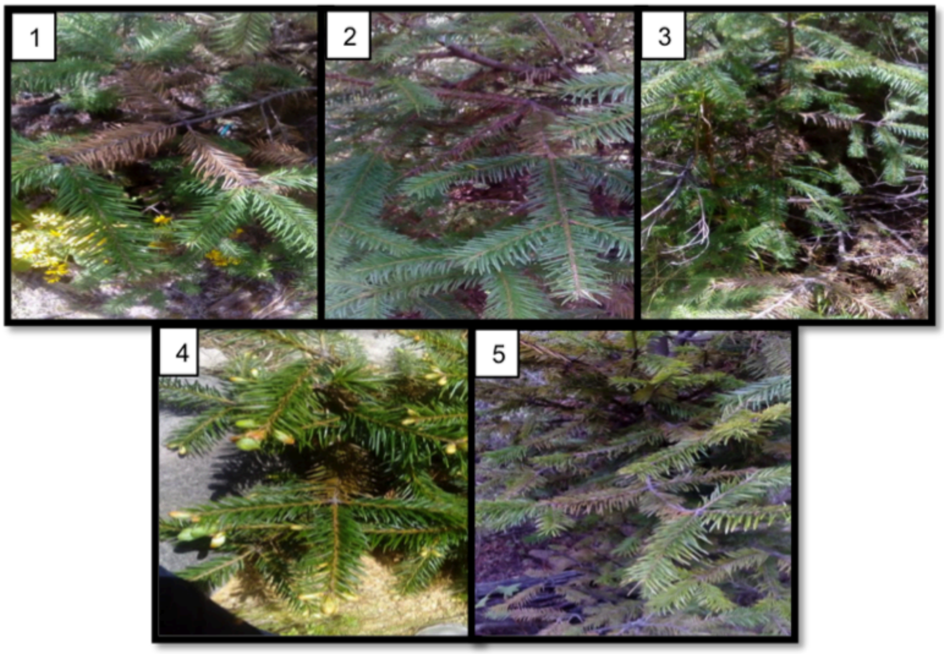

Figure S1 Photographs of the branches for each sampled sacred fir tree. **(a)** asymptomatic trees **(b)** symptomatic trees.

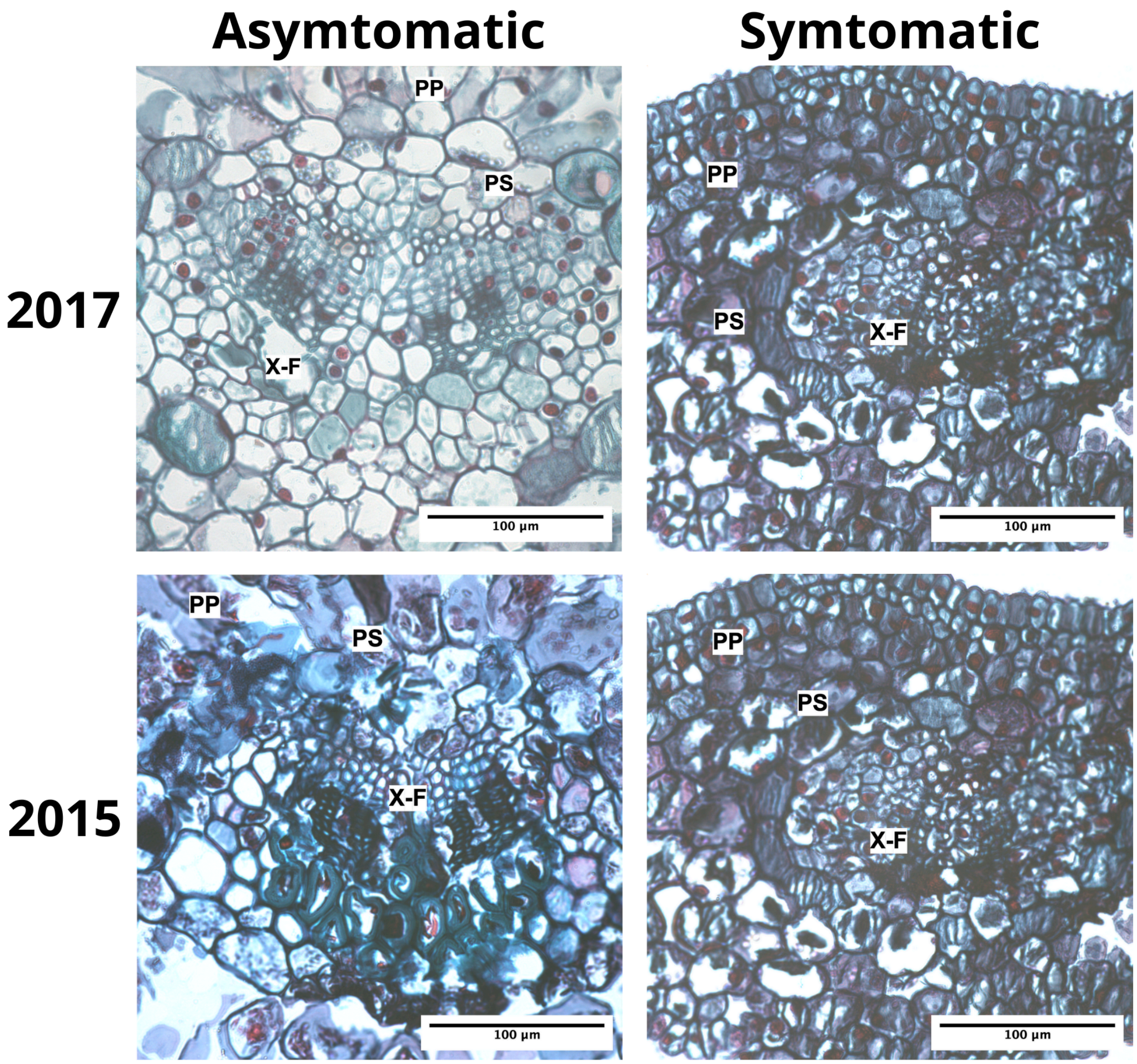

Figure S2 Histological sections of needles from asymptomatic (left) and symptomatic (right) sacred fir (*Abies religiosa*) individuals from two growing seasons (2017 top; 2015 bottom). All bars = 10µm. PP, palisade parenchyma; SP, spongy parenchyma; X-P, xylem and phloem.

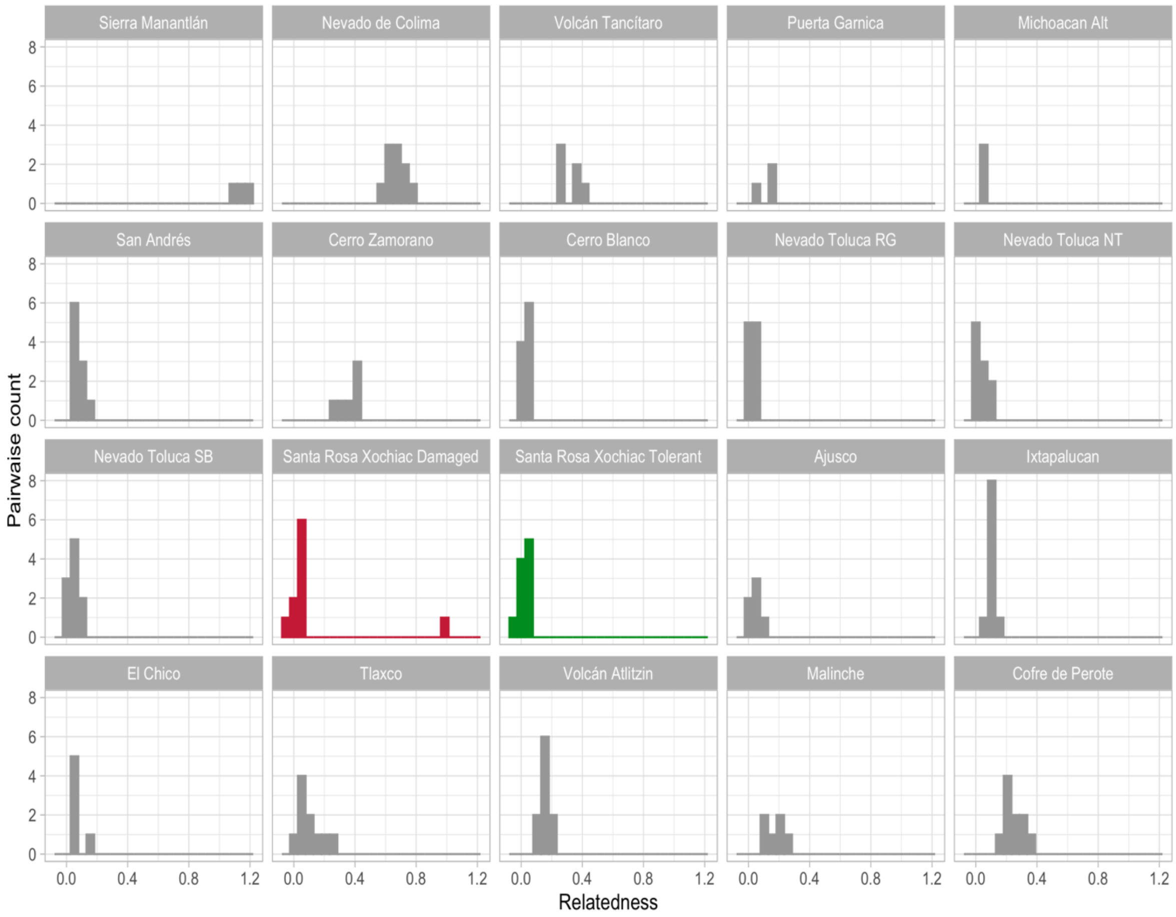

Figure S3 Relatedness between sacred fir (*Abies religiosa*) individuals used for genetic assignment analyses. Asymptomatic individuals from study sites in green, symptomatic trees in red.
