# Supplementary material for: Histologic, metabolomic, and transcriptomic differences in fir trees from a peri‐urban forest under chronic ozone exposure
Source: Ecol Evol. 2024 May 13;14(5):e11343. doi: 10.1002/ece3.11343 (PMC11091488; doi:10.1002/ece3.11343)
Supplement: Supplementary file 1 — Appendices S1‐S9 [file ECE3-14-e11343-s001.zip › TABLES_Abies_EcologyEvolution.docx.pdf]

TABLES

Table S1 Differentially expressed transcripts in symptomatic vs asymptomatic sacred fir (*Abies religiosa*)

Contig ID	Log_2 fold change[a]	Query length, nts	Score[b] / Max query cover in the 1st 5 hits, %	Annotation	Notes
AB_000588_T.1	7.257	707	L / 40	Hypothetical protein KI387_017072, partial [*Taxus chinensis*]	The only hit returned by the ncbi Blastx
AB_045531_T.1	4.450	1192	M / 68	Hypothetical protein	Mostly bacterial hits
AB_015092_T.1	1.614	1944	H / 89	*Nuclear fusion defective 4-like, Nodulin-like*	*Nuclear fusion defective 4* in *A. thaliana* is involved in response to salt stress (Sottosanto et al. 2007).
AB_036475_T.1	1.437	650	H / 78	*Chitinase* class VII / II / IV / or EP3-like / 4 / 5	Chitinases are involved in responses to various abiotic and biotic stresses.<br>An acidic chitinase is **over-regulated after ozone exposure** in tobacco (Ernst et al. 1992)
AB_018867_T.1	1.302	409	L / 37	Unknown [*Picea sitchensis*]	Four hits in 2 unknown proteins of *P. sitchensis* (Could be conifer-specific protein)
AB_029334_T.1	-1.187	2594	H / 72	*Probable L-type lectin-domain containing receptor kinase S.5*	L-type lectin receptor kinases are involved in defense response to bacteria and oomycetes (Bouwmeester and Govers 2009).
AB_029013_T.1	-1.371	1214	VL / 21	Hypothetical protein	Three hits in two different OFRs

AB_035458_T.1<br><br>AB_038616_T.1	-2.8306<br><br>- 4.951	928<br><br>752	H / 99<br><br>H / 88	*Leucine-rich repeat (LRR) receptor-like serine/threonine protein kinase*	A large family of LRR receptor-like kinases (RLK) participate in all aspects of plant development, in response to abiotic stresses, in defense processes and in plant-microbe interactions.<br>Loss of the LRR-RLK GHR1 **resulted in O3 sensitivity** in *A. thaliana*, likely mediated by the associated disruption of stomatal function (Sierla et al. 2018).
AB_027319_T.1	-7.549	895	L / 39	Tetratricopeptide repeat (TPR)-like / patatin-like phospholipase domain protein / oidium resistance required protein 1/ TOM1-like protein 2	Members of TPR protein superfamily includes ones with potential to interact with Hsp90/Hsp70 as co-chaperones in nucleus and cytoplasm, thus participating in response to biotic stresses; RNA binding proteins involved in mRNA edition in plastid and mitochondria, are involved in plant development.<br>Patatin-like phospholipase domain proteins involved in plant development, synthesis of secondary metabolites, cell death, defense responses, response to abiotic stresses (Lebeda et al. 2014).

AB_038562_T.1	-23.104	951	No hit	No hit	No significant similarity either in BLASTn search in NCBI nr database, neither in congenie.

[a] Positive value: up regulated in symptomatic trees; Negative value: down regulated in symptomatic trees;
[b] H: high (>200); M: medium (80-200); L: low (50-80); VL: very low (40-50).

Table S2 Differentially expressed transcripts in symptomatic sacred fir trees during high vs moderate O_3 concentration periods.

**ID Locus**	**Log_2 fold change[a]**	**Query length, nts**	**Score[b] / Max query cover in the 1st 5 hits, %**	**Annotation**	**Notes**
AB_002157_T.1	4.255	609	VL / 30	Hypothetical protein [*Acinetobacter baumannii*]	NCBI BLASTn returns five hits of mRNA sequences of *Picea glauca* with 81.49% to 87.93% identity
AB_028063_T.1	3.717	1034	No hit	No hit	
AB_029211_T.1	3.265	1193	H / 44<br><br>(H / 76)	No Apical Meristem, (NAC) transcription factor (Unannotated protein [*Picea sitchensis*])	Members of the huge family of NAC transcription factors are involved in many aspects of plant development, defense response to bacteria and other organisms, response to water deprivation and to abscisic acid, secondary metabolic processes. ANAC013, ANAC016, ANAC017, ANAC053 and ANAC078 regulate oxidative stress in *A. thaliana* (De Clercq et al. 2013).
AB_023740_T.1	2.911	1320	H / 62	Xyloglucan endotrans glucosylase (XET) /hydrolase; Glycosyl hydrolase family 16	XET enzymes participate in cell wall remodeling, thus modulating its expansion and strength.<br>The contig covers complete XET CDS.<br><br>Expression of XET coding gene XTR9 **increased in response to O_3** (Zhang et al. 2017).

AB_015079_T.1	2.094	1291	M / 24	Linker histone H1	Linker (H1) histones are the most variable histones; H1.3 variant of *A. thaliana* is involved in adaptive responses to abiotic stress (Rutowicz et al. 2015).
AB_008838_T.1	-1.7	1312	H / 89	UDP-glucosyl transferase (UGT) 7-deoxyloganetin glucosyltransferase	The enzymes of the UGT family act on a variety of substrates and participates in many metabolic processes, including flavonol (e.g. UGT78D1/At1g30530), tetrapyrrole (e.g UGT85A1/AT1G22400) or terpenoid (e.g. UGT89B1/ AT1G73880) biosynthesis. Some UGTs involved in response to abiotic and biotic stresses (Rehman et al. 2018). Transcription of UGT78D2/At5g17050 gene was **decreased after O_3 exposure** for 2 days (Booker et al. 2012).

[a] Positive value: up regulated during high O_3 concentration periods; Negative value: down regulated during high O_3 concentration periods;
[b] H: high (>200); M: medium (80-200); L: low (50-80); VL: very low (40-50).

Table S3 Differentially expressed transcripts in asymptomatic sacred fir trees during high vs. moderate O_3 concentration periods**.**

**ID Locus**	**Log_2 fold change[a]**	**Query length, nts**	**Score[b] / Max query cover in the 1st 5 hits, %**	**Annotation**	**Notes**
AB_010244_T.1	7.274	2007	H / 59	Metal tolerance protein (MTP) 5, 11 Cation diffusion facilitator (CDF) efflux family proteín	Plant MTPs from CDF family are involved in enhancing resistance to heavy metal tolerance
AB_022453_T.1	6.398	613	M / 56	Pathogenesis-related (PR) thaumatin family protein	PR thaumatin family proteins are involved in defense response, response to fungus, to osmotic stress, to water deprivation, to wounding, regulation of metabolism and plant development (e.g. AT4G36010 and AT1G20030 in *A. thaliana*).
AB_040533_T.1	6.07	561	H / 90	Disease resistance-responsive dirigent-like protein	Many dirigent-like proteins are involved in defense response; some in response to wounding, cell wall biogenesis and metabolic processes.
AB_025629_T.1	5.388	1582	H, M / 88	LRR and NB-ARC domain disease resistance protein; disease resistance protein RPP13,	NB-ARC domain disease resistance (R) proteins in plants are involved in pathogen recognition and subsequent activation of innate immune responses. Besides, Glyma12g01420 was **upregulated in response to elevated ozone** in Glycine max (Leisner et al.

				RPM1, RGA2, RGA4	2014).
AB_022256_T.1	4.635	1436	H / 82<br>--	S-adenosyl methionine (SAM) synthase	Small family of plant S-adenosylmethionine synthases, or methionine adenosyltransferase (MAT) produces SAM from methionine and ATP. Methyl group of SAM can be transferred to a variety of molecules that includes nucleic acids, proteins, lipids and secondary metabolites. Therefore, the methylation rates for a variety of substrates affects multiple aspects of plant fitness. Besides, in plants SAM is a precursor of ethylene and polyamines. Histone and DNA methylation is highly important for the regulation of gene expression (Sekula et al. 2020).
AB_013716_T.1	3.549	1989	H / 74	3-ketoacyl (oxoacyl)-CoA synthase	Members of the 3-ketoacyl-CoA synthase family are involved in the biosynthesis of very long chain fatty acids (VLCFA), therefore, in cuticle development and wax and suberin synthesis. They also have an important role in response to cold, to light stimulus, to osmotic stress and to wounding
AB_043005_T.1	3.549	1193	M / 63	B-box-type Zinc finger and CCT domain protein CONSTANS-LIKE (COL)	COL transcription factors are involved in regulation of plant growth and development, control of flowering time and responses to stresses (Khatun et al. 2021).
AB_000610_T.1	3.054	1461	H / 68	beta-1,3-glucanase, or glucan endo-1,3-beta-glucosidase	Beta-1,3-glucanases degrade plant callose and components of plant, fungi and bacteria cell walls, therefore, are involved in defense response. Some of them are also involved in

					response to cold, heat and wounding.
AB_021997_T.1	2.999	2144	H / 81	Isocitrate lyase/ Phosphoenolpyruvate phosphomutase	Isocitrate lyase is a glyoxylate cycle enzyme; it is involved in plant salt tolerance (Yuenyong et al. 2019).
AB_002147_T.1	2.926	1211	H / 82	Peroxidase 72 class III peroxidase	*A. thaliana* Peroxidase 72 (AT5G66390) is involved in lignin biosynthesis and in response to oxidative stress; many class III peroxidases are located in cell wall and involved in cell wall modification; some may play a role in generating H_2O_2 during defense response. **Near-ambient concentrations of ozone can induce ascorbate peroxidase** APX1 gene expression in *A. thaliana* and tobacco (Kubo et al. 1995, Wang et al. 1999). At least part of the induction of heat shock proteins during light stress in Arabidopsis is mediated by H_2O_2 that is scavenged by APX1.
AB_000596_T.1	2.883	475	No hit	No hit	
AB_013152_T.1	1.832	1494	H / 65	Carboxylesterase 15; alpha/beta hydrolase fold	Carboxylesterases hydrolyze esters of short-chain fatty acids and involved in metabolism of jasmonic acid and salicylic acid and in systemic acquired resistance. They belong to the larger alpha/beta hydrolase fold superfamily of enzymes.
AB_028624_T.1	1.798	967	H / 40	Early nodulin-like (ENODL) with cupredoxin/ plastocyanin domain	Cupredoxins contain type I copper centers and are involved in inter-molecular electron transfer reactions. ENODLs extracellular proteins are anchored in the plasma membrane.

					AtENODL1 (AT5G53870) transcript is up-regulated in leaves of *A. thaliana* subjected to a combination of drought and heat stress. AtENODL2 (AT4G27520) is involved on responses to water deprivation, abscisic acid, salt stress, light and temperature stimuli (Rizhsky et al. 2004).
AB_031334_T.1	1.736	752	M / 40	Zinc finger Ran-binding domain-containing protein 2; RNA-binding protein c17h9.04c; UPF0481 protein	Mammalian zinc finger Ran-binding domain-containing protein 2 is an RNA-binding protein involved in alternative splicing.
AB_015079_T.1	1.73	1291	M / 24	Histone H1	Linker (H1) histones are the most variable histones; H1.3 variant of *A. thaliana* is involved in adaptive responses to abiotic stress (Rutowicz et al. 2015).
AB_039330_T.1	1.601	974	L (M) / 25	Hypothetical protein (plants), Set1 complex component ash2	The Set1 complex specifically methylates Lys-4 of histone H3 (H3K4). H3K4me is an epigenetic modification involved in the **regulation (induction) of gene expression.**
AB_013119_T.1	1.429	465	No hit		Two Picea NCBI BLASTn hits suggest that it could be conifer-specific polyA RNA.
AB_018867_T.1	-1.431	409	L / 37	Unknown protein [*Picea sitchensis* only]	Could represent a conifer-specific protein
AB_000811_T.1	-1.949	1592	H / 61	Flavonol synthase	Some 2OG-Fe(II) oxygenases (as AT5G24530

				2OG-Fe(II) oxygenase GA2ox9, GA2ox10	in *A. thaliana*) participates in flavonoid biosynthesis; therefore, they may be involved in response to salicylic acid and defense response to bacteria, oomycetes and fungus.<br>A homology to GA2ox9 that contribute to cold stress tolerance and involved in response to water deprivation and wounding (Lange et al. 2020), is also revealed.
AB_029470_T.1<br><br>AB_008960_T.1	-3.459<br><br>-5.169	1182<br><br>1226	H / 69<br><br>H / 80	(Iso)eugenol synthase 1, isoflavone reductase, propenylphenol synthase 1<br>NmrA-like protein<br>NAD(P)H-binding<br>NAD dependent epimerase/dehydratase family	The inferred proteins possess similarity to several classes of enzymes with Rossman fold. Among them are the isoflavone reductases involved in response to oxidative stress and to wounding, as well as the propenylphenol synthases involved in synthesis of phenylpropanoid compounds, propenyl-phenols (Wibe et al. 1997), presumed to serve mainly in defense against herbivores and parasites.
AB_000071_T.1	-6.206	1408	H / 60	Ferritin, desiccation-related protein PCC13-62	Arabidopsis ferritins are essential to protect cells against oxidative damage (Ravet et al. 2009).

[a] Positive value: up regulated during high O_3 concentration periods; Negative value: down regulated during high O_3 concentration periods;
[b] H: high (>200); M: medium (80-200); L: low (50-80); VL: very low (40-50).

Table S4 Wilcoxon Test. Interactions between Condition (asymptomatic or symptomatic), Needle age (2015 or 2016) and Period (high or moderate).

	Period moderate 87 ppb		Period high 170 ppb	
	**Metabolite**	**Sig.**	**Metabolite**	**Sig.**
**Condition** Asymptomatic - Symptomatic	α-caryophyllene	0.0004871**	α-caryophyllene	N.S.
	α-Cubebene	0.007197*	α-Cubebene	N.S.
	β-Caryophyllene	0.0001299**	β-Caryophyllene	N.S.
	β-Cubebene	0.004525*	β-Cubebene	N.S.
	β-Pinene	0.0004871**	β-Pinene	N.S.
	δ-Cadinene	0.0007253**	δ-Cadinene	N.S.
	Bornyl acetate	0.0115*	Bornyl acetate	N.S.
**Needle age** one-year and two-years exposition	α-caryophyllene	N.S.	α-caryophyllene	N.S.
	α-Cubebene	N.S.	α-Cubebene	N.S.
	β-Caryophyllene	N.S.	β-Caryophyllene	N.S.
	β-Cubebene	N.S.	β-Cubebene	N.S.
	β-Pinene	N.S.	β-Pinene	N.S.
	δ-Cadinene	N.S.	δ-Cadinene	N.S.
	Bornyl acetate	N.S.	Bornyl acetate	N.S.

	**Metabolite**	**Sig.**
**Period** 87ppb - 170 ppb	α-caryophyllene	0.001953*
	α-Cubebene	0.003906*
	β-Caryophyllene	0.001953*
	β-Cubebene	0.003906*
	β-Pinene	0.001953*
	δ-Cadinene	0.005859*
	Bornyl acetate	0.001953*

(***) Significant at the 0.0001 probability level. (**) Significant at the 0.001 probability level. (*) Significant at the 0.05 probability level. (.) Significant at the 0.1 probability level. (ns) nonsignificant.

Table S5 Number of genes mapped for each sample.

Tree condition	O3 concentration period	ID sample	Number of genes identified as expressed**	Number of genes with no reads mapped*
Asymptomatic	high	Asymptomatic 1	37,601	0
		Asymptomatic 2	33,200	4,401
		Asymptomatic 3	34,182	3,419
		Asymptomatic 4	34,840	2,761
		Asymptomatic 5	33,366	4,235
	moderate	Asymptomatic 1	35,460	2,141
		Asymptomatic 2	34,256	3,345
		Asymptomatic 4	35,031	2,570
symptomatic	high	Symptomatic 1	34,048	3,553
		Symptomatic 2	33,983	3,618
		Symptomatic 3	34,060	3,541
		Symptomatic 4	33,663	3,938
		Symptomatic 5	33,981	3,620
	moderate	Symptomatic 1	35,738	1,863
		Symptomatic 2	35,020	2,581
		Symptomatic 5	34,293	3,308

***Number of genes with no reads mapped**: refers to genes without any reads mapped to the reference transcriptome of *A. balsamea*, considering the total number of mapped genes.

** **Number of genes identified as expressed:** refers to genes with reads mapped to the reference transcriptome of *A. balsamea.*

Table S6 RNA-seq data per sample.

Sample	Total reads	Mapped	Mapped %	Properly paired	Properly paired %	Singletons	Singletons %
Asymptomatic 1	26628465	25110645	94.30%	23207744	87.79%	190570	0.72%
Asymptomatic 2	29394389	27421473	93.29%	25506062	87.47%	216864	0.74%
Asymptomatic 3	28885822	26935913	93.25%	25005412	87.24%	206331	0.72%
Asymptomatic 4	27148620	24890979	91.68%	23160294	85.90%	190051	0.70%
Asymptomatic 5	25402180	22810050	89.80%	21279266	84.36%	153044	0.61%
Asymptomatic 1	86373044	80384008	93.07%	74602376	87.09%	601512	0.70%
Asymptomatic 2	39848295	36957834	92.75%	34301814	86.78%	271419	0.69%
Asymptomatic 4	30581813	28117524	91.94%	26128276	86.06%	188559	0.62%
Symptomatic 1	29917209	26626122	89%	24575346	82.81%	204199	0.69%
Symptomatic 2	20519755	19680381	95.91%	18198494	89.39%	124258	0.61%
Symptomatic 3	34920801	33514452	95.97%	30677044	88.59%	257139	0.74%
Symptomatic 4	33932229	30786857	90.76%	28520838	84.73%	245596	0.73%
Symptomatic 5	34662281	32472479	93.68%	30328610	88.12%	230530	0.67%
Symptomatic 1	29755812	25145836	84.51%	23338336	79.07%	219234	0.74%
Symptomatic 2	32034433	29891742	93.31%	27696704	87.09%	228013	0.72%
Symptomatic 5	39785361	35702980	89.74%	32688214	82.84%	330867	0.84%

Supplementary references

Booker F, Burkey K, Morgan P, Fiscus E, Jones A (2012) Minimal influence of G-protein null mutations on ozone-induced changes in gene expression, foliar injury, gas exchange and peroxidase activity in Arabidopsis thaliana L.: Minimal influence of G-proteins on ozone responses. Plant Cell Environ 35:668–681.

Bouwmeester K, Govers F (2009) Arabidopsis L-type lectin receptor kinases: phylogeny, classification, and expression profiles. J Exp Bot 60:4383–4396.

De Clercq I, Vermeirssen V, Van Aken O, Vandepoele K, Murcha MW, Law SR, Inzé A, Ng S, Ivanova A, Rombaut D, van de Cotte B, Jaspers P, Van de Peer Y, Kangasjärvi J, Whelan J, Van Breusegem F (2013) The Membrane-Bound NAC Transcription Factor ANAC013 Functions in Mitochondrial Retrograde Regulation of the Oxidative Stress Response in Arabidopsis. Plant Cell 25:3472–3490.

Ernst D, Schraudner M, Langebartels C, Sandermann H (1992) Ozone-induced changes of mRNA levels of β-1,3-glucanase, chitinase and 'pathogenesis-related' protein 1b in tobacco plants. Plant Mol Biol 20:673–682.

Khatun K, Debnath S, Robin AHK, Wai AH, Nath UK, Lee D-J, Kim C-K, Chung M-Y (2021) Genome-wide identification, genomic organization, and expression profiling of the CONSTANS-like (COL) gene family in petunia under multiple stresses. BMC Genomics 22:727.

Kubo A, Saji H, Tanaka K, Kondo N (1995) Expression of arabidopsis cytosolic ascorbate peroxidase gene in response to ozone or sulfur dioxide. Plant Mol Biol 29:479–489.

Lange T, Krämer C, Pimenta Lange MJ (2020) The Class III Gibberellin 2-Oxidases AtGA2ox9 and AtGA2ox10 Contribute to Cold Stress Tolerance and Fertility. Plant Physiol 184:478–486.

Lebeda A, Mieslerová B, Petřivalský M, Luhová L, Špundová M, Sedlářová M, Nožková-Hlaváčková V, Pink DAC (2014) Resistance mechanisms of wild tomato germplasm to infection of Oidium neolycopersici. Eur J Plant Pathol 138:569–596.

Leisner CP, Ming R, Ainsworth EA (2014) Distinct transcriptional profiles of ozone stress in soybean (Glycine max) flowers and pods. :13.

Ravet K, Touraine B, Boucherez J, Briat J-F, Gaymard F, Cellier F (2009) Ferritins control interaction between iron homeostasis and oxidative stress in Arabidopsis. Plant J 57:400–412.

Rehman HM, Nawaz MA, Shah ZH, Ludwig-Müller J, Chung G, Ahmad MQ, Yang SH, Lee SI (2018) Comparative genomic and transcriptomic analyses of Family-1 UDP glycosyltransferase in three Brassica species and Arabidopsis indicates stress-responsive regulation. Sci Rep 8:1875.

Rizhsky L, Liang H, Shuman J, Shulaev V, Davletova S, Mittler R (2004) When defense pathways collide. The response of Arabidopsis to a combination of drought and heat stress. Plant Physiol 134:1683–1696.

Rutowicz K, Puzio M, Halibart-Puzio J, Lirski M, Kroteń MA, Kotliński M, Kniżewski Ł, Lange B, Muszewska A, Śniegowska-Świerk K, Kościelniak J, Iwanicka-Nowicka R, Żmuda K, Buza K, Janowiak F, Jõesaar I, Laskowska-Kaszub K, Fogtman A, Zielenkiewicz P, Tiuryn J, Kollist H, Siedlecki P, Ginalski K, Świeżewski S, Koblowska M, Archacki R, Wilczyński B, Rapacz M, Jerzmanowski A (2015) A specialized histone H1 variant is

required for adaptive responses to complex abiotic stress and related DNA methylation in Arabidopsis. Plant Physiol:pp.00493.2015.
Sekula B, Ruszkowski M, Dauter Z (2020) S-adenosylmethionine synthases in plants: Structural characterization of type I and II isoenzymes from Arabidopsis thaliana and Medicago truncatula. Int J Biol Macromol 151:554–565.
Sierla M, Hõrak H, Overmyer K, Waszczak C, Yarmolinsky D, Maierhofer T, Vainonen JP, Salojärvi J, Denessiouk K, Laanemets K, Tõldsepp K, Vahisalu T, Gauthier A, Puukko T, Paulin L, Auvinen P, Geiger D, Hedrich R, Kollist H, Kangasjärvi J (2018) The Receptor-like Pseudokinase GHR1 Is Required for Stomatal Closure. Plant Cell 30:2813–2837.
Sottosanto JB, Saranga Y, Blumwald E (2007) Impact of AtNHX1, a vacuolar Na+/H+ antiporter, upon gene expression during short- and long-term salt stress in Arabidopsis thaliana. BMC Plant Biol 7:18.
Wang J, Zhang H, Allen RD (1999) Overexpression of an Arabidopsis Peroxisomal Ascorbate Peroxidase Gene in Tobacco Increases Protection Against Oxidative Stressl. :8.
Wibe A, Borg-Karlson A-K, Norin T, Mustaparta H (1997) Identification of plant volatiles activating single receptor neurons in the pine weevil (Hylobius abietis). J Comp Physiol A 180:585–595.
Yuenyong W, Sirikantaramas S, Qu L-J, Buaboocha T (2019) Isocitrate lyase plays important roles in plant salt tolerance. BMC Plant Biol 19:472.
Zhang L, Xu B, Wu T, Wen M, Fan L, Feng Z, Paoletti E (2017) Transcriptomic analysis of Pak Choi under acute ozone exposure revealed regulatory mechanism against ozone stress. BMC Plant Biol 17:236.
